# Emotion regulation in children (ERiC): A protocol for a randomised clinical trial to evaluate the clinical and cost effectiveness of Mentalization Based Treatment (MBT) vs Treatment as Usual for school-age children with mixed emotional and behavioural difficulties

**DOI:** 10.1371/journal.pone.0289503

**Published:** 2023-08-17

**Authors:** Nick Midgley, Rose Mortimer, Mark Carter, Polly Casey, Lisa Coffman, Julian Edbrooke-Childs, Chloe Edridge, Peter Fonagy, Manuel Gomes, Anoushka Kapoor, Susannah Marks, Peter Martin, Bettina Moltrecht, Emma Morris, Nikola Pokorna, Tara McFarquhar

**Affiliations:** 1 The Anna Freud Centre, London, United Kingdom; 2 University College London, United Kingdom; 3 Barnet, Enfield and Haringey NHS Trust, United Kingdom; 4 Barnet Parent/Carer Forum, United Kingdom; 5 Oxford Health NHS Foundation Trust, United Kingdom; 6 University of East Anglia, Norwich, United Kingdom; PLoS ONE, UNITED STATES

## Abstract

**Background:**

The majority of children referred to Child and Adolescent Mental Health Services (CAMHS) in the UK will present with mixed emotional and behavioural difficulties, but most mental health treatments are developed for single disorders. There is a need for research on treatments that are helpful for these mixed difficulties, especially for school-age children. Emotion Regulation (ER) difficulties present across a wide range of mental health disorders and mentalizing may help with regulation. The ability to mentalize one’s own experiences and those of others plays a key role in coping with stress, regulation of emotions, and the formation of stable relationships. Mentalization Based Therapy (MBT) is a well-evidenced therapy that aims to promote mentalization, which in turn increases ER capacities, leading to decreased emotional and behavioural difficulties. The aim of this study is to test the clinical- and cost-effectiveness of MBT compared to treatment as usual for school age children with emotional and behavioural difficulties. If effective, we hope this approach can become available to the growing number of children presenting to mental health services with a mix of emotional and behavioural difficulties.

**Materials and methods:**

Children referred to CAMHS aged 6–12 with mixed mental health problems (emotional and behavioural) as primary problem can take part with their parent/carers. Children will be randomly allocated to receive either MBT or treatment as usual (TAU) within the CAMHS clinic they have been referred to. MBT will be 6–8 sessions offered fortnightly and can flexibly include different family members. TAU is likely to include CBT, parenting groups, and/or children’s social skills groups. Parent/carers and children will be asked to complete outcome assessments (questionnaires and tasks) online at the start of treatment, mid treatment (8 weeks), end of treatment (16 weeks) and at follow up (40 weeks).

**Trial registration:**

**Clinical trial registration:** ISRCTN 11620914.

## Introduction

In 2021, one in six (16.0%) children in England aged 5 to 16 were identified as having a probable mental disorder [1]. The majority of these children, when presenting to child mental health services, do not receive an evidence-based treatment. This is partly because most empirically tested treatments were developed to treat single disorders [2, 3], whereas the evidence indicates that the structure of mental health symptoms does not straightforwardly map onto traditional diagnostic categories and comorbidity of mental health problems is high [4, 5]. Indeed, most children referred for treatment show high rates of transdiagnostic co-morbidity–this is the rule, not the exception [6]. As a result, there is a significant research-practice gap.

A solution to this research-practice gap is for clinical trials to focus on transdiagnostic interventions that target causal and maintaining factors underlying a range of clinical presentations. Transdiagnostic interventions are especially suitable for children, who show a great overlap in symptoms and a high level of transitory symptoms across developmental stages [7–9]. Transdiagnostic interventions also have good potential for implementation, as they allow clinicians to address diverse diagnoses and sub-threshold symptoms within a single treatment model, and simultaneously target multiple problem areas, so limiting training burden on clinicians [9–11].

Because of the ubiquity of emotion regulation (ER) difficulties across a wide range of psychopathologies, research has identified ER as a core transdiagnostic mechanism [12]. ER refers to implicit and explicit processes and strategies involved in regulating emotional states. Both adequate down-regulation of negative emotional states and up-regulation of positive states during childhood play a critical role in adaptive development and well-being [13]. ER difficulties have been associated with risk of psychopathology across both emotional and behavioural disorders [14–16]. Findings of a recent meta-analysis suggest that interventions can enhance ER in youth, and that these changes correlate with improvements in psychopathology [17]. However, of the 21 studies in the meta-analysis, only 4 targeted children aged 6–12, thereby highlighting a significant evidence gap for this age group. In addition, most research and interventions have neglected the role of positive/adaptive ER strategies, despite recent research highlighting their importance, especially for children and young people [17].

The capacity to ‘mentalize’ is now recognised as a key component of positive or adaptive ER [18]. Mentalizing has been defined as ‘the process by which we make sense of each other and ourselves, implicitly and explicitly, in terms of subjective states and mental processes’ [19] (p. 11). The ability to mentalize one’s own experiences and those of others plays a key role in coping with stress, regulation of emotions, and the formation of stable relationships [18]. Empirical studies have shown that deficits in the capacity to mentalize are predictive of maladaptive ER in both clinical and non-clinical populations [20, 21], and that there are deficits in mentalizing capacity among children with either internalizing or externalizing psychopathology [22–25].

The importance of parental mentalizing to the well-being of children is also clearly established. Parental mentalizing has been found to be significantly associated with both child’s internalizing and externalizing symptoms and social–emotional competencies [26]. The quality of a carer’s mentalizing is positively associated with sensitive caregiving, strengthened parent-child relationships, and secure attachment [27, 28]. Parents with higher mentalizing capacity are better able to tolerate emotional distress in their children, which is thought to be helpful in managing parenting stress [29]. A systematic review exploring the association between parental mentalizing and children’s mental health outcomes [30] highlighted that poor maternal mentalization is associated with overcontrolling parenting [31], a higher incidence of child anxiety [32], child emotion regulation difficulties [33] and greater child externalizing behaviours [34].

Mentalization Based Treatment (MBT) was originally developed as a treatment for adults with a diagnosis of borderline personality disorder [19], where emotion dysregulation is a key feature. The evidence base for its effectiveness with this group is good [35]. More recently it has been adapted for therapeutic work with children and families [36]. For school age children, there is preliminary evidence that promoting a child’s mentalizing capacity can improve ER and symptomatic improvement, including children’s behavioural and emotional difficulties [24, 37]. Moreover, improvement in parents’ capacity for mentalizing has been associated with reductions in children’s internalizing and externalizing problems [38]. However, as a recent systematic review has highlighted, high quality clinical trials of MBT for school-age children are lacking [39], and time-limited MBT has yet to be evaluated as a transdiagnostic intervention for school-age children.

In summary, given that many children presenting to mental health services experience comorbid difficulties, there is a need for evidence-based transdiagnostic interventions that effectively target mechanisms underlying co-occurring mental health difficulties; ER is the best-evidenced mechanism implicated in a range of common mental health disorders. MBT is a well-evidenced therapy for a range of populations that aims to promote mentalization, which in turn increases ER capacities, leading to decreased emotional and behavioural difficulties. Until now, children under 12 have not had the benefit of this transdiagnostic approach, as the evidence for its effectiveness in this population has not yet been examined. The aim of this study is to test the clinical- and cost-effectiveness of MBT against treatment as usual (TAU) for school-age children with comorbid internalizing and externalizing difficulties. We will conduct the first randomised controlled trial of MBT for this population and examine the role of ER as a mediator of treatment response. Our hypotheses are as follows:

Hypothesis 1: Children allocated to MBT will experience a significantly greater reduction in mental health problems–both internalizing and externalizing–when compared with the TAU group.
Hypothesis 2: Families allocated to MBT will also experience a greater improvement in a range of secondary outcomes, including improved capacity for emotion regulation, and decreased parental stress, and a reduction in health and social service use and costs compared to the TAU group.
Hypothesis 3: The impact of treatment on child mental health will be mediated, in part, by changes in capacity for emotion regulation (in both parent and child).

The Implementation and Process Evaluation (IPE) aims to investigate: a) model fidelity, b) the experience of MBT (including the change process) from the perspective of service users; and c) any barriers to implementation and scalability post-trial.

If effective, we hope this scalable, transdiagnostic approach can become available to the growing number of children presenting to mental health services with a mix of internalizing and externalizing difficulties. In addition, it may enable a more efficient allocation of health and social care resources by reducing the need for longer-term, more intensive specialist mental health interventions.

## Materials and methods

### Trial design

This study will be a pragmatic, individually randomized, superiority trial comparing MBT with TAU, with those administering outcome measures and analysing the data, blind to group assignment. An internal pilot will inform the optimal delivery of the main study, which will be a fully powered, randomised control trial of the intervention. Both the internal pilot and main trial will have an embedded qualitative component. Assessments of capacity for ER will enable mediator analyses to identify mechanisms underlying symptom change.

The internal pilot will take place before the main RCT, across two NHS Trusts, following the same recruitment and data collection procedures as the main trial. The internal pilot will be an opportunity to test a) the process and procedures of training and supervising the practitioners delivering MBT; b) the recruitment processes (including screening of referrals and contact with families); and c) the feasibility of data collection processes. With regard to this third aim, this will include feasibility of remotely conducting two task-based assessments of emotion regulation. Recent recommendations for the assessment of emotion regulation processes suggest that researchers adopt a multi-modal approach, where self-report measures are complemented with task-based and/or observational measures, although few studies have done this sufficiently especially as part of clinical trials [17]. Hence, one of our objectives for the internal pilot phase is to explore the feasibility of our multi-modal assessment approach as part of a clinical trial and test the convergence validity of the different ER measures. If successful, we will carry forward self-report and one or both task-based ER measures to the main trial where they may be employed at baseline only.

### Study setting

The study will be set in two large mental health trusts in the UK: Barnet, Enfield and Haringey (BEH) Mental Health NHS Trust and Oxford Health NHS Trust, which between them run 12 child and adolescent mental health services (CAMHS). Involvement with the study is supported at senior management level within these CAMHS. These services receive over 3,000 referrals of children aged 6–12 per year, from a diverse cultural and social background. The services are multi-disciplinary, including clinical psychologists, child psychiatrists, child psychotherapists, family therapists, social workers, primary mental health workers and clinical nurses.

### Participants

320 children aged 6–12 with mixed (internalizing and externalizing) mental health problems, and their parents/carers. Children will be recruited from referrals to participating CAMH services.

Based on data available from services, approximately 60% of referrals are accepted as suitable for CAMHS, and a case file review has indicated that approximately 40% of accepted cases are likely to meet our inclusion criteria, with the majority of other referrals in this age group related to Autistic Spectrum Disorder (ASD) or neurodevelopmental disorders. This would make an estimated eligible pool of 720 children per year. We anticipate a consent rate of approximately 40–50%. The number of children included in each participating CAMHS team will be monitored by the trial team throughout the recruitment phase of the study. If the intake of children falls behind recruitment targets, appropriate action will be taken, including the possibility of adding additional clinical sites to the study.

During the internal pilot we will aim to recruit 40 families to the study (20 to MBT, 20 to Treatment as Usual). The research team will monitor recruitment during the pilot phase in order to identify any barriers to recruitment and to allow for any changes in procedure.

### Eligibility criteria

#### Inclusion criteria

Child aged 6–12; carer-reported Strengths and Difficulties Questionnaire (SDQ) total difficulties score for child of ≥14 with emotional problems ≥5 and conduct score ≥3; combined with a functional impairment score of ≥1.

#### Exclusion criteria

Current participation in another mental health intervention trial. A pre-existing clinical diagnosis (in child or parent) of: psychotic disorder, ASD, pervasive developmental disorder, eating disorder, severe learning difficulty, or (in parent) severe substance abuse disorder. Children will also be excluded where the referring clinician identifies an immediate risk of harm to self or others.

### Recruitment, screening and gaining informed consent

**Fig 1:**
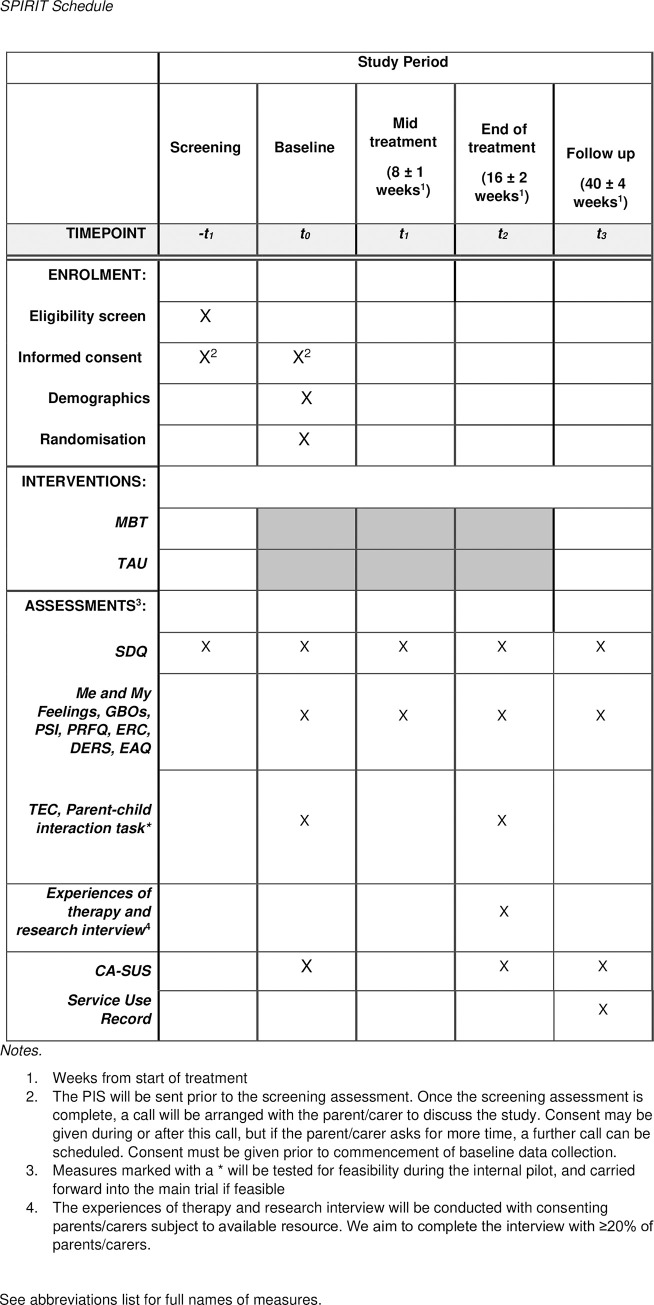
SPIRIT schedule of enrolment, interventions, and assessments.

**Fig 2:**
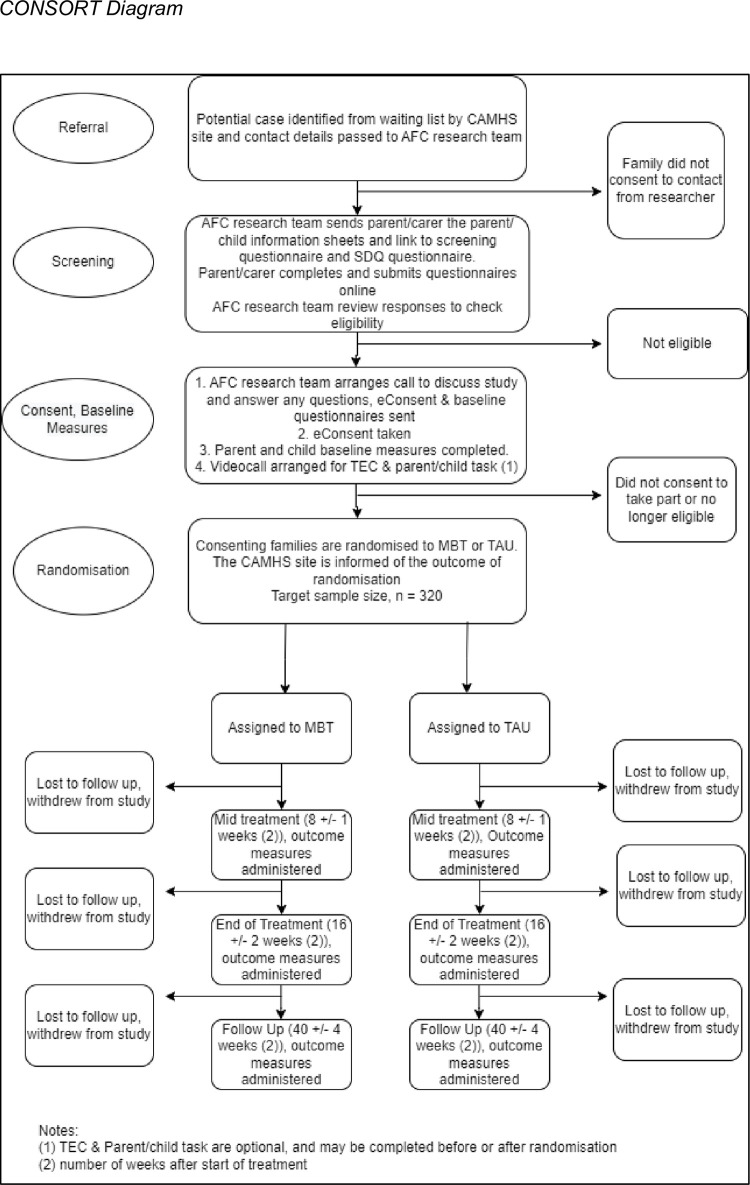
CONSORT diagram.

The study began recruitment in April 2023 and will recruit participants over a 16-month period (4 months for the internal pilot, and 12 months for the main trial), with the pilot sites continuing to recruit while data from the internal pilot is analysed and sites are set up for the main trial. A SPIRIT schedule of enrolment, interventions, and assessments is presented in Fig 1, and participants’ journey through the trial is mapped out in Fig 2, and specific aspects are explained in more detail below.

All new referrals will be initially screened by a member of the CAMHS intake team to see if the child will be aged 6–12 by the time of randomisation and that there are no indications that any exclusion criteria apply. The parents/carers of those children who may be eligible will be offered further information about the study; consent will be sought from the family to pass the parent’s contact details to the research team. The research team will then send information sheets by email to those parents/carers who have agreed to share their contact details, along with a link to a screening questionnaire. If the screening questionnaire indicates that the child is eligible, the research team will offer a telephone/video call to discuss the study further and answer any questions the parent/carer may have. If the family is interested in the study, the parent/carer will be sent a link to the eConsent form. Children will be invited to provide eAssent to participate at study entry, and verbal assent will be sought by researchers prior to commencing optional interviews and tasks.

Once informed consent has been granted, the parent/carer will be able to access and complete the parent/carer and child baseline questionnaires online. At the family’s request, all measures can also be completed on a call with the research team while accessing a blank PDF version of the questionnaires. A video call with the family will be arranged for completion of the Test of Emotional Comprehension (TEC) task and parent-child interaction task, if agreed to in eConsent form.

### Randomization

Once mandatory elements of baseline data collection have been completed, participants will be randomized to either MBT or TAU, stratified by CAMH service and age group (6–9 years and 10–12 years old), using permuted blocks of size 2 within each stratum. The randomization sequence will be generated from random numbers generated in the R software for statistical computing [40] and pre-loaded into the REDCap data management platform, which will be used for data collection.

The randomization sequence will be stored but concealed within the REDCap data capture system. When all information relevant for stratification (CAMH service and age) has been completed, a researcher has the option to initialize randomization. Only then will the allocation be revealed within the REDCap database, and the CAMH service will be informed of the result of the randomization, so that treatment can be initiated.

### Interventions

Interventions in both arms of the study will be delivered by therapists working in the CAMHS teams taking part in the study. As part of study set up, all therapists taking part in the study will be randomly selected to either deliver MBT or treatment as usual. Therapists will be stratified by NHS banding, which takes into consideration profession and level of experience, to ensure that any systematic differences between groups are due to the model of intervention. Therapists who have had previous training in MBT will by default be allocated to the MBT arm of the study.

#### MBT

**Fig 3:**
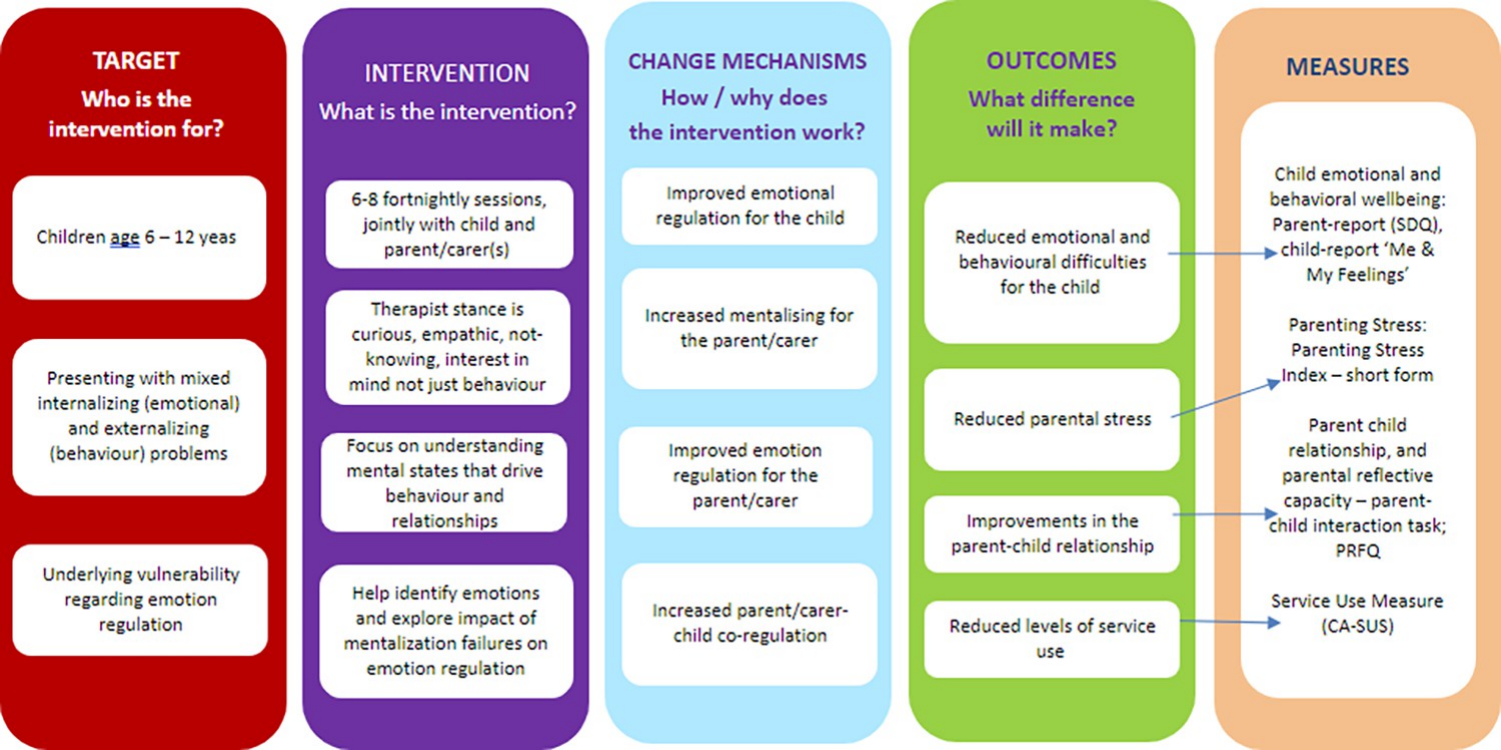
MBT logic model.

The adaptation of MBT used in this study is a manualized, transdiagnostic model designed for children aged 6–12 and their carers. It aims to promote adaptive ER by attending to the capacity to understand oneself and others in terms of underlying mental states. MBT consists of 6–8 sessions, delivered fortnightly, which can flexibly involve different members of the family, with a primary focus on promoting mentalizing and emotion regulation in the parent-child relationship (see Fig 3). Therapists randomly allocated to deliver treatment for the intervention group will receive a 3-day MBT training, plus fortnightly group supervision to support treatment fidelity. Practitioners are supported by an interactive online treatment manual and the use of a self-completed MBT Fidelity Scale (https://manuals.annafreud.org/mbt-c/index.html).

#### Treatment as Usual (TAU)

The control group will be offered TAU. As there is no single evidence-based treatment for this group, and because practice is not standardised across CAMH services in England, TAU is likely to include CBT, parenting groups, and/or children’s social skills groups. Scoping work with CAMH services has confirmed that 6–8 fortnightly sessions would be the usual length of intervention for this client group. Therapists will be provided with usual supervision, in line with existing practice in participating CAMHS teams. As part of the study, more detailed data about TAU will be collected, including type of intervention, treatment intensity and professionals offering treatment.

### Discontinuation and withdrawal

Children and families can withdraw from either treatment or the trial without further explanation at any point. Where children or families withdraw from treatment, they will be invited to continue to participate in the trial. Reasons for discontinuing or withdrawal from the trial will be recorded, where families agree to provide such a reason. If a child requires more intensive care, such as inpatient hospitalization, the participant can be exited from the treatment, if this is clinically indicated, but will be encouraged to remain part of the study.

### Data collection

There will be three types of data collected: questionnaires, online tasks (subject to testing of feasibility during the internal pilot phase) and a semi-structured interview.

Questionnaire data will be collected using a secure, browser-based web application for developing, maintaining, and managing different types of surveys and securing online/offline data collection (REDCap). Participants will have the option to self-complete these questionnaires by following a link to the REDCap webpage, or to complete them via video or telephone call with a member of the research team. If collected during the video call, the responses to questionnaires will not be video recorded. One questionnaire (Service Use Record) will be completed by the participant’s local CAMHS team.

#### There are two tasks

A parent-child interaction task, and a child task (the Test of Emotional Comprehension; TEC). These interactive tasks will take place during a video call between the family and a member of the research team and part of the video call will be recorded. The child’s responses to the questions in the TEC task will be collected in real-time during the video call and entered directly into REDCap by the member of the research team conducting the call. The parent-child interaction task is facilitated by the member of the research team and the video call recorded; the recording is saved securely and later analysed and scored. All data entered directly into REDCap is securely stored with UCL’s Data Safe Haven.

The semi-structured interviews are part of the implementation and process evaluation (see Outcome Measures). They will take place after treatment has ended during an online call and audio recorded by the research team. The recording will be saved securely at on Anna Freud Centre (AFC) secure servers and transcribed and analysed.

For families who do not have access to the technology needed to facilitate a video call, data collection will take place over a telephone call, or else families will be given the opportunity to access the necessary technology at the CAMHS where treatment is taking place. In order to ensure that recruitment is inclusive, interpreters will be made use of to support data collection, where required.

The use of REDCap automatically scores and transmits the data, reducing the risk of data entry error. All research assistants are trained on the administering of the outcome measures. A summary of data collection timepoints is provided in the schedule of enrolment, interventions, and assessments (SPIRIT schedule) (Fig 1).

Participants’ personal data will be securely stored and only accessible by members of the ERiC research team at AFC. At the end of the study, data will be archived in a safe and secure location.

### Blinding

Parent/carers and children in the trial will not be blinded to intervention group. However, research assistants and those involved in data collection and analysis (including coding of ER tasks) will be blind to intervention group. Trial Managers will initiate the randomisation of participants and the outcome of the randomisation will be sent directly to staff at the CAMHS sites (see section ‘Randomisation’).

### Outcome measures

Brief, well-validated tasks and questionnaires will be administered at four points: baseline, 8 weeks after start of treatment (mid-treatment), 16 weeks after start of treatment (end of treatment) and 40 weeks after start of treatment (follow-up). An overview of all assessment points and outcomes are detailed in the SPIRIT (Fig 1).

In order to maximize clinical validity, data will be gathered through multiple methods (including questionnaire, interview and observational assessments) and from multiple perspectives (including self- and carer-reports). A consensus-based standard set of international measures [41] has been used. All questionnaire measures selected have established reliability, validity, utility/practicability and acceptability for this age group; they have also been reviewed by our parent co-applicants to ensure that they are acceptable, relevant and culturally appropriate. The pilot phase of the study will be used to test the feasibility of online, task-based assessment of parent-child ER capacity and mentalizing.

Primary outcome measure: Parent-rated Strengths and Difficulties Questionnaire (SDQ) Total Difficulties Score [42]. The SDQ is one of the most widely used mental health measures for children in the UK, covering both emotional (anxiety and depression) and behavioural (ADHD and conduct) symptoms.

Secondary outcome measures will include a range of carer- and child-report assessments and tasks to assess:

1. Emotional and behavioural problems,
  - Me and My Feelings [43, 44], a brief 16-item self-report measure for children, including both emotional and behavioural sub-scales.
2. Personalized treatment goals,
  - Goal Based Outcomes (parent/carer-defined) [45], a personalized goal-based measure completed by parent/carers.
3. Parenting stress,
  - Parental Stress Index–Short Form [46, 47], a 36-item parent/carer-report measure of stress and the parent-child relationship.
4. Parental mentalizing capacity,
  - Parental Reflective Functioning Questionnaire [48], an 18-item parent/carer-report measure of parental mentalizing.
5. Service use and costs.
  - Child and Adolescent Service Use Schedule (CA-SUS) [49], a questionnaire which records service use 3 months prior to baseline, during the course of the treatment, and between end of treatment and follow-up
  - Service Use Record, a questionnaire completed by CAMHS team to record the services offered and attended between baseline and end of treatment, and end of treatment and follow up.
6. Emotion regulation in child and parent will be assessed using the following:
  - Emotional Awareness Questionnaire [50], a 30-item child self-report measure of emotional awareness and mentalizing capacity.
  - Test of Emotional Comprehension (TEC) [51], an online task to assess child’s capacity for emotional understanding, ER and mentalizing capacity. This task takes approximately 15 minutes to complete. The test is divided into a set of stories in an established order. The test booklet consists of illustrations with a story that is read for each situation. On every page, there are four possible outcomes represented by emotional facial expressions. Throughout the test, there are five options to choose from: happy, sad, angry, afraid, alright. The children are asked to assign an emotion represented by a facial expression to the situation. A digitalised version of the test booklet will be used in the current study. Feasibility of its use will be tested in the internal pilot study.
  - Emotion Regulation Checklist for Children [52], a 24-item parent/carer-report measure of child’s ER capacity.
  - Difficulties in Emotion Regulation Scale [53], a 36-item parent/carer report measure of parent/carer’s ER capacity.
  - Parent-Child Interaction Task, a parent/carer-child discussion task to assess capacity of parent/carer to support ER and mentalizing in the parent/carer-child relationship, and to assess child’s use of ER strategies. The researcher asks the child to recall a time when they felt sad, and to talk to the parent/carer about what happened and how it made them feel. After discussion, the interviewer asks the child: “What did you do to make your sad feelings go away?” followed by one additional prompt to elicit further details e.g., “What else did you do?”. This is repeated for three other emotions: happy, afraid, angry. The task is video and audio recorded. The task is adapted from Davis et al. [54] and Shipman and Zeman [55] and takes approximately 15 minutes to complete; the feasibility of its use will be tested in the internal pilot study. The coding scheme developed by Davis et al. [54] will be used to identify the child’s use of emotion regulation strategies. An adapted version of the coding scheme developed by Vanwoerden [56] will be used to assesses degree of positive mentalizing, and degree of negative or maladaptive mentalizing (hyper or hypo mentalizing) within the dyad.
7. Treatment fidelity
  Treatment fidelity will be assessed using the supervisor-report version of the MBT (Child) Fidelity Scale (Malberg, Bate and Midgley [Unpublished]), which can also be used to secondarily examine how fidelity to the MBT model relates to treatment outcome.
8. Implementation and Process Evaluation:
  - The Experiences of Therapy and Research Interview (Midgley, Ansaldo, Parkinson, Holmes, Stapley and Target [Unpublished]), a semi-structured interview carried out with parents and (where possible) the child, exploring the experience of change, the therapy process and of participating in research.
  - Online survey–examining stakeholder views on the scalability of the MBT intervention at the end of the delivery phase.

### Monitoring of potential harm

To date there is a lack of agreement on suitable approaches to assessing and monitoring potential harm in the context of clinical trials for psychological treatments [57], especially in the context of child and family treatments. For the purposes of this study, we will adopt a broad definition of a Serious Adverse Event (sa). These include (in child or carer):

- Violent behaviour resulting in serious physical harm to another person
- Family relationship breakdown
- New or escalated self-harm
- New or escalated suicidal ideation (a preoccupation with suicide/thoughts about suicide, with no clear plans to take own life)
- Suicidal intent (concrete and deliberate plans to end own life, with a conscious desire to escape from the world and a resolve to act purposively in this regard, e.g., a suicide attempt. This may be a deliberate action or disclosing of a deliberate action)
- Other life-threatening events
- Hospitalization due to drugs or alcohol, self-harm, or for psychiatric reasons (including, in-patient hospitalization, or significant disability/incapacity)
- Death, including suicide

SAEs may occur during the course of being involved in the trial. This could be irrespective of intervention or data collection or may be related to the intervention or data collection. Ascertaining a relationship between an SAE and the intervention in the trial must be assessed on a case-by-case basis. The individual identifying the SAE will therefore complete an SAE form, which contains an assessment of relatedness. Any SAEs considered to be ‘likely related’ to the intervention will be reviewed with clinical leads in the CAMHS team where the SAE has been identified and will qualify for expedited reporting to the ERiC Trial Steering Committee (TSC), who are responsible for monitoring the study. SAEs which are both related and unexpected will also be reported to the Research Ethics Committee (REC). Expected occurrences in the trial include the potential for some distress for participants as part of exploring painful experiences as part of therapy. For further information on SAEs, please refer the ERiC SAE procedure document.

### Internal pilot

During the internal pilot, 40 children and families will be recruited in a sub-sample of CAMHS teams. During this phase any potential barriers to training, recruitment, intervention delivery or data collection will be identified. Towards the end of the delivery phase, a problem-solving workshop will be held in each site included in the pilot phase of the trial, attended by trial therapists, service managers and any other relevant CAMHS staff to gain feedback on training, recruitment, delivery of intervention, and supervision procedures. Any issues arising, as well as potential adaptations, will be discussed in meetings of the Trial Management Group and the TSC, both during and upon completion of the internal pilot. Any recommended changes will be implemented for the full trial. The MBT training team will also review levels of model fidelity during the pilot phase, including any items on the scale which had the lowest mean ratings, and any adaptations to training and/or supervision that should be made will be identified, to improve model fidelity in the main study.

The internal pilot will also be an opportunity to examine alternative approaches to the assessment of emotion regulation. Although multiple measures exist to assess ER in children, most clinical trials do not include the perspectives of both parents/carers and children. Moreover, recent research has highlighted the importance of assessing different components of ER if we are to understand how ER relates to psychopathology, and which aspects of ER need to be targeted in interventions. Due to the primarily deficit-focused approach in clinical psychology, the field has long neglected the role of adaptive ER, especially in youth populations. This is of particular importance, because recent evidence shows that the lack of adaptive–but not presence of maladaptive—ER was significantly associated with psychopathology for children with emotional disorders [58]. The pilot phase of this trial will therefore test the feasibility of evaluating different elements of ER, including self- and carer-reported assessment alongside task- and observation-based approaches. This will allow us to explore how different ER components relate to different symptom clusters and examine which aspects of ER change due to MBT treatment. If task- and observation-based approaches are shown to be feasible, and there is indication that they add an additional perspective to the assessment of ER, including co-regulation in the parent-child relationship, they will be included in the full trial.

Data collected in the internal pilot will be used in the analysis for the full RCT: participants recruited in the pilot will be included within the total number of participants for the full trial.

### Statistical plan and data analysis for main trial

#### Sample size and power calculation

The total sample size for the study will be 320. The power analysis was informed by an analysis of 71,763 UK CAMHS clinical records held by the Child Outcomes Research Consortium (CORC, https://www.corc.uk.net/), as well as by published psychotherapy trials in CAMHS settings. CORC data suggests that children meeting our inclusion criteria have a mean SDQ Total Difficulties score of 24.6 at presentation to CAMHS, with a standard deviation of 5.0. This score is our primary outcome. We assumed that the smallest clinically meaningful post-treatment difference on the SDQ is 1.5 points, which is equivalent to a standardised treatment effect difference of 0.3. The power analysis aimed to establish the number of children per therapist to give at least 80% power to detect this effect at the 5% significance level using a two-sided test. The statistical model will account for clustering of children within therapists, and of therapists within the CAMHS, via random intercepts. We will adjust for baseline SDQ scores.

We calculated the required sample size based on published formulae [59] and made the following assumptions: The within-patient correlation was assumed to be within-patient = 0.6, which is close to the median value of 0.59 estimated by a meta-analysis of within-patient correlations [60]. We estimated that 40 therapists will participate in the study and assumed a therapist effect of ICC therapist = 0.02. We think this ICC is conservative. There is little data on therapist ICCs in CAMHS settings specifically, but the recent Improving Mood with Psychoanalytic and Cognitive Therapies (IMPACT) trial, set in English CAMHS, reported ICC < 0.01 [61]. A smaller therapist ICC would increase power. The power analysis assumed that outcomes do not vary by CAMHS, which is also conservative. If there was intra-site correlation, power would increase, since each service would act as its own control. Given these assumptions, and allowing for 15% loss to follow-up, the total required sample size is *N* = 320 (160 children per treatment group, 8 children per therapist). The calculated power under this design is 80.8%.

### Statistical data analysis plan for the main trial

The primary analysis aims to establish the difference between the MBT and TAU groups in the SDQ Total Difficulties score at the end of treatment (16 weeks, Hypothesis 1). Children are clustered within therapists, who in turn are clustered within CAMH services. The primary analysis model is a linear mixed effects model, with random intercepts for therapists and services, controlling for SDQ and age group at baseline. The evidence for a difference in SDQ scores after treatment will be assessed via a two-sided t-test of the coefficient of the treatment indicator variable, using a parametric bootstrap to estimate standard errors. A 5% significance level will be used as a cut-off to support a finding of superiority of MBT over TAU.

Analogous models will be used to assess the evidence regarding a difference between the MBT and TAU groups with respect to secondary outcomes (Hypothesis 2).

To investigate potential mediation of the effect of MBT on child mental health via improvement in emotion regulation (Hypothesis 3), we will conduct a mediation analysis using treatment allocation as the exposure, the Emotion Awareness Questionnaire (EAQ) score as the mediator, and the SDQ Total Difficulties Score as the outcome. We will additionally adjust for both EAQ and SDQ scores at baseline. Summary scores from the validated scales will be used, rather than latent variable scores, in order to maintain consistency with the effect estimates of the primary analysis. We will conduct an analogous mediation analysis using parental emotion regulation, assessed by the Difficulties in Emotion Regulation Scale (DERS). More complex mediation models will be developed using exploratory analyses.

To examine whether the improvement in child SDQ Total Difficulties is clinically significant at the individual level, the reliable change index [62] will be calculated for each participant using the SDQ scores at baseline and post-intervention. Children will be classified as recovered, improved, unchanged, or deteriorated. We will compare the distribution of this classification in the MBT and TAU groups using an ordinal logistic regression model.

We will also estimate the difference in treatment effect between MBT and TAU on SDQ Total Difficulties and all secondary outcomes at 40-week follow-up, using statistical models analogous to those for the primary analysis.

All primary and secondary analyses will be carried out as intention-to-treat analyses. If some values at the primary endpoint are missing, the following strategy will be employed: 1) Evaluate likely processes of missingness and assess their potential for causing bias; 2) Conduct a complete cases analysis as the primary analysis; (3) Conduct information-anchored sensitivity analyses using controlled multiple imputation under MNAR assumptions to gauge the sensitivity of the trial results to potential violations in the MAR/MCAR assumptions [63, 64].

### Economic evaluation

The aim of the economic analysis will be to provide an assessment of the relative costs and benefits of MBT compared to TAU over the 40-week follow-up period. The study perspective will be that of the NHS and personal social services sectors, including those provided within the education sector. Health and social care service use will be collected using the CA-SUS questionnaire at baseline (covering the last 3 months) and at the 16- and 40-week follow-up points (covering the period since the last assessment). In addition, a Service Use Record will be completed by the CAMHS team at follow up to record the services offered and attended during the trial period. Collecting resource use at baseline will better characterise what ‘treatment as usual’ involves in terms of health care resources and allow for an adjustment for any potential baseline imbalances. Service use will be valued using PSSRU unit costs of health and social care [65] and national tariffs (NHS Reference Costs). MBT and TAU sessions will be costed using a bottom-up approach and using data on staff use (contacts). We will adopt a cost-consequences analysis (CCA) to report value for money of MBT versus TAU at 40 weeks. The CCA will report a disaggregated summary of all the costs and benefits of MBT versus TAU, without attempting to combine them into a single measure such as a cost-effectiveness or cost-utility ratio. This allows decision makers to decide which costs and benefits are most relevant to their decision context, and whether the relevant benefits are worth any additional costs that might be incurred. We will report mean costs and consequences, together with 95% confidence intervals, by treatment groups at baseline, 16 and 40 weeks. The consequences of interest for the CCA will be: SDQ and a range of carer and child-reported measures: The range of costs to be reported: intervention, medication, primary care, hospital care, social care, school-based service use. Costs and consequences will not be discounted given the short follow-up period.

### Implementation and process evaluation

#### Treatment fidelity

Fidelity to the MBT model will be assessed using the MBT Fidelity Scale (Malberg, Bate and Midgley [Unpublished]), completed by supervisors.

#### Experience of therapy and the change process from the service user perspective

In order to examine the experience of therapy and the change process, parents/carers and children who have agreed to an optional interview in the consent form will be interviewed at the end of intervention (16 weeks), using an adapted version of the Experiences of Therapy and Research Interview (Midgley, Ansaldo, Parkinson, Holmes, Stapley and Target [Unpublished]),to explore their experiences of therapy and the change process. We aim to interview at least 20% of parents/carers and (where possible) children. The interviews will explore parents/carers’ and children’s experiences of the intervention offered, how acceptable they found it, and any facilitators or barriers they found in engaging with the programme. For those who stopped attending the MBT, or who had low attendance, there will be an exploration of reasons for stopping/non-attendance, and of potential barriers to participation. Interviews will be audio-recorded and transcribed. Interviews with children may include the use of drawings, to enable a richer understanding of their experiences of therapy.

Interviews with children and parents/carers will be transcribed, and the data analysed using Framework Analysis [66], a qualitative method which allows both a priori issues and emergent data-driven themes to guide the development of the analytic process. Drawings, where included, will be incorporated at two stages of the analysis, using a specific qualitative analysis method used previously by members of the research team [67].

#### Implementation and potential for scalability

A link to an online survey will be emailed to all stakeholders in the CAMHS taking part (practitioners, administrative staff, and managers) towards the end of the intervention phase to gain their views on the scalability of the MBT intervention at the end of the delivery phase of the full RCT. Once the online survey is completed, a single problem-solving workshop, including members of the research team, and selected practitioners, administrative staff, and managers, will be organised, in order to review the findings of the survey and discuss potential ways in which any identified barriers to post-trial implementation could be overcome, if the intervention is demonstrated to be effective.

### Trial oversight

A Trial Manager will oversee day-to-day management of the trial, with two research assistants. Trial staff will also include a statistician, a health economist, a clinical lead (with responsibility for training and ongoing supervision of CAMHS clinicians) and an expert by experience who will review all procedures and materials, Chair the Parent/Carer Advisory Group and help to problem solve operational issues. The principal and associated investigators will oversee all study operations. Clinical management will be provided by Leads in each CAMHS Trust. A Trial Management Committee will meet weekly to manage the day-to-day running of the trial (chief investigator, trial managers, research assistants, and site leads). A TSC will meet twice a year to provide oversight to ensure that the project is conducted according to the UK’s Research Governance Framework. A Data Monitoring and Ethics Committee (DMEC), comprised of experts who are independent of the sponsor (Anna Freud), will oversee progression of the data collection and undertake regular monitoring of the MBT intervention arm for signs of deterioration. This will be done by reviewing items on measures identified as markers of deterioration in the child. Unlike the TSC, the DMEC will have access to unblinded, comparative data. DMEC meetings will take place six-monthly, where required, once data collection has commenced, usually two weeks prior to TSC meetings in order for them to report to the TSC.

### User involvement

A parent co-applicant and an Advisory Group of parents/carers and young people with experiences of child mental health services will continue to inform study design and delivery, specifically: a) recruitment of research staff; b) selection of outcome measures; c) ensuring information sheets and consent forms are designed with consideration of the needs of families; d) designing data management systems that have the confidence of families (timing of assessments, settings, confidentiality); e) co-developing structured qualitative interviews; and f) dissemination to public.

### Ethical considerations

Ethical approval was granted by the NHS Health Research Authority and necessary local permissions, and information governance approvals, will be obtained before patient recruitment. The research will be conducted in line with relevant ethical guidelines, including the Declaration of Helsinki. Voluntary and fully informed eConsent will be obtained from parents/carers and informed assent from children. Families will be given Information Sheets (parent and child versions) in age-appropriate language, co-developed in collaboration with the Young Champions group at the AFC, as well as with the parent/carer representatives. Families will have the right to withdraw from the study at any point, without needing to give a reason. Their decisions concerning participation will not in any way affect their access to the usual support and treatment in CAMHS. Data will be anonymized, and personal details stored securely in accordance with General Data Protection Regulations 2018 (GDPR). Safety and child protection concerns will be closely monitored in line with safeguarding procedures. The TSC will monitor the safety of participants and child protection concerns. Where safeguarding concerns are identified, the CI and local site lead will be immediately informed, and the CI will also inform the local safeguarding oversight group (SOG). Any safeguarding issues will be reported to the TSC, ethics committee, and funder.

### Trial registration

The trial has been registered with the International Standard Randomised Controlled Trial Number (ISRCTN) registry (11620914).

### Data protection

A data protection impact assessment has been completed and agreed by the Data Protection Officer at the AFC. All participants will receive a data privacy notice alongside the study information sheet, in line with GDPR regulations. Access to the trial dataset will be covered by the collaboration agreement between the trial partner organisations. Only certain members of the CORE research team will have access to identifiable information about participants (NM, JEC, BM, CE, PC, AK and NP).

## Discussion

One of the main barriers to children receiving evidence-based interventions in CAMHS is the lack of research evaluating transdiagnostic treatments that target mechanisms underlying the mixed internalizing and externalizing difficulties experienced by many young people. Research is often organised around specific diagnoses, but these children do not fit easily within diagnostic frameworks, meaning that there is a lack of evidence-based interventions available for them.

This trial will address this research-practice gap. Developed in collaboration with experts by experience and service providers, this study will be the first full-scale, sufficiently powered clinical trial to evaluate MBT as an intervention for school-age children with mixed emotional and behavioural difficulties. By targeting an underlying mechanism (ER), which has been shown to play a role in a range of mental health difficulties, it has the potential to identify a brief, manualized treatment that may be effective for a significant proportion of school-aged children referred to mental health services. MBT can be delivered by a range of mental health professionals in a real-world setting, mapping on well to the transdiagnostic THRIVE model used to organise child mental health services across the UK and internationally [68].

Further, the qualitative component of this research will seek to identify what makes MBT ‘therapeutic’ from the perspective of families. Interviews will explore young people and parent/carers’ understanding of what makes treatment effective, why, and the role of ER in this process. This will provide valuable insights, informing our understanding of which treatments work for whom, and under what conditions [69].

This study is innovative in the use of multiple measures to assess different components of ER, integrating the perspectives of the child and the parent/carer. This will allow us to explore how different ER components relate to different symptom clusters and examine which aspects of ER change due to MBT treatment. Furthermore, most research focuses on ER within the individual, but for school-age children ER happens mostly in relationship to others; by examining parent-child co-regulation of emotions, this study represents a significant methodological advancement. In this way, this study is aligned with the “Roadmap for Mental Health Research in Europe” [70], in that it seeks to identify and interrogate putative mechanisms of change.

Many studies evaluating treatments in child mental health settings are limited by a lack of follow-up [71]. This study is ambitious in that it will include a six-month follow-up post-intervention. If MBT proves to be superior to TAU in the first 40 weeks, we will ask families for consent to be re-contacted, so that a long-term follow-up study can answer the question of whether the beneficial effects are maintained two and five years from baseline.

The Medical Research Council guidance on evaluation of complex interventions [72] emphasises the importance of including a process evaluation and an economic evaluation within clinical trials. This study is ambitious in including both. The health-economic evaluation will improve our understanding of the economic implications, and value for money, of implementing MBT compared to current treatment options. In doing so, the trial will better map and evaluate ‘treatment as usual’ for a group of children who are poorly served by current evidence-based treatment guidelines, as they do not fit easily within existing diagnostic frameworks. The process evaluation will help to explain discrepancies between expected and observed outcomes, to understand how context influences outcomes, and to provide insights to aid implementation. Taken together, the health-economic and process evaluation will ensure that the findings of this evaluation are relevant and actionable for decision-makers, ensuring real world impact for children and families.

It is well established that child mental health is strongly associated with levels of deprivation and poverty [73] and the Covid-19 pandemic is likely to have further increased inequalities in mental health. The Centre for Mental Health predicts that up to 1.5 million children and young people in the UK aged under 18 will need new or additional mental health support as a direct consequence of the crisis [74]. Developing effective interventions for these children and families may help to mitigate health inequalities across the lifespan, hopefully preventing difficulties from becoming entrenched and persisting into adulthood. In evaluating MBT for children aged 6–12, the project will address the UN Sustainable Development Goal: ‘ensure healthy lives and promote well-being for all at all ages.’

### Dissemination

The findings of the study will be disseminated to the academic and scientific community via conference presentations and publication of findings in leading peer-review journals.

Working with an Advisory Group of parents/carers, we will create and publish a video summary of the full trial results and an infographic which will be disseminated through our social media and Learning Network of almost 30,000 allied professionals, enabling children and families to be better informed about evidence-based treatment models, and to empower practitioners to learn about the study findings. We will also create briefing notes which will be shared with policy makers.

If MBT is shown to be clinically- and cost-effective, dissemination into practice will be facilitated by the AFC’s track-record of developing and delivering training programmes to mental health practitioners, including the development of Child Wellbeing Practitioners (CWP), as well as providing guidance to healthcare managers and commissioners of services, and to national and international bodies responsible for developing clinical guidelines (e.g., NICE). Training materials are already translated into several languages, and a web-based MBT treatment manual allows for rapid dissemination of training. There are clear pathways for practitioners who are trained during the study to go on to become supervisors themselves, thus embedding ownership and sustainability into the study design.

The UK’s long-term plan for the NHS promises that 100% of children and young people who need specialist care can access it within a decade, but there is a deficit in evidence-based models that can be delivered by a wide range of professionals to children with a broad range of presenting difficulties. If this study demonstrates that primary mental health workers can be trained to deliver effective MBT, then this approach has the potential to become central to meeting the government’s ambitions for child mental health services.

### Trial status

This trial began recruitment in April 2023.

This manuscript is based in protocol version 2.

## Supporting information

S1 Checklist SPIRIT 2013 checklist: Recommended items to address in a clinical trial protocol and related documents*. (DOC)

S1 File (PDF)

S2 File (PDF)
