# Supplementary material for: Emotion regulation in children (ERiC): A protocol for a randomised clinical trial to evaluate the clinical and cost effectiveness of Mentalization Based Treatment (MBT) vs Treatment as Usual for school-age children with mixed emotional and behavioural difficulties
Source: PLoS One. 2023 Aug 17;18(8):e0289503. doi: 10.1371/journal.pone.0289503 (PMC10434917; doi:10.1371/journal.pone.0289503)
Supplement: S1 File — (PDF) [file pone.0289503.s002.pdf]

Emotion Regulation in Children (ERiC): A Randomised Clinical Trial to Evaluate the Clinical and Cost Effectiveness of Mentalization Based Treatment (MBT) vs Treatment as Usual for School-Age Children with Mixed Emotional and Behavioural Difficulties

Protocol v2.0 05.12.2022

Principal investigator	Professor Nick Midgley
Trial team contact	eric@annafreud.org
Protocol author(s)	Nick Midgley, Rose Mortimer, Mark Carter, Polly Casey, Lisa Coffman, Julian Edbrooke-Childs, Chloe Edridge, Peter Fonagy, Manuel Gomes, Anoushka Kapoor, Susannah Marks, Peter Martin, Bettina Moltrecht, Emma Morris, Nikola Pakorna and Tara McFarquhar
Sponsor	The Anna Freud Centre will provide administrative, scientific and financial support to the study as the hosting institution. It will also provide the infrastructure to carry out the study and access to their networks of policymakers, therapists and researchers.
Funder	The Kavli Trust, grant number Kavli2021-0000000090
IRAS ID	316392
ISRCTN number	ISRCTN11620914
Trial type	Randomised Control Trial
Participants	320 children aged 6-12, and their carers
Number and type of sites	Two NHS Trusts, between them providing up to 12 Child and Adolescent Mental Health Services (CAMHS) clinics
Primary outcome	The primary outcome will be parent/carer-rated Strengths and Difficulties Questionnaire (SDQ), Total Difficulties Score (Goodman, 2001)
Secondary outcomes	Secondary outcomes will include a range of parent/carer- and child-report assessments and tasks to assess:<br>1) emotional and behavioural problems,<br>2) personalized treatment goals,<br>3) parenting stress,<br>4) emotion regulation and mentalizing, and<br>5) service use and costs.<br><br>Me and My Feelings (Deighton et al., 2013; Patalay et al., 2014) – a brief self-report measure for children of mental health, including both emotional and behavioural subscales;<br><br>Goal Based Outcomes (parent/carer-defined; Law & Jacob, 2015)- a personalized goal-based measure completed by carers;<br><br>Parental Stress Index (Abidin, 2012; Haskett et al., 2006) – a parent-report measure of stress and the parent-child relationship;<br><br>Parental Reflective Functioning Questionnaire (Luyten et al., 2017) – a parent/carer-report measure of parental mentalizing;<br><br>Emotional Awareness Questionnaire (Rieffe et al., 2008) – brief child self-report measure of emotional awareness and mentalizing capacity;<br><br>Test of Emotional Comprehension (Pons & Harris, 2000) – web-based tasks to assess child's capacity for emotional understanding, emotion regulation, and mentalizing capacity;

	Emotion Regulation Checklist for Children (Shields & Cicchetti, 1997) parent/carer-report measure of child's emotion regulation capacity;<br><br>Difficulties in Emotion Regulation Scale (Gratz & Roemer, 2004)– parent/carer report measure of parent's emotion regulation capacity;<br><br>Parent-Child Interaction Task (adapted from: Davis et al., 2010; Shipman & Zeman, 1999; Vanwoerden, 2020) – to assess capacity of parent/carer to support ER and mentalizing in the parent-child relationship, and to assess child's use of emotion regulation strategies<br><br>Treatment fidelity will be assessed using a supervisor-report version of the MBT (Child) Fidelity Scale. (Malberg et al., 2019)<br><br>Qualitative component: A sub-sample of 64 (20%) parent/carers and children from both arms of the study will be interviewed at end of intervention, using an adapted version of the Experiences of Therapy and Research Interview (Midgley et al., 2011), to explore their experiences of therapy and the change process.<br><br>Health economic analysis: We will undertake a cost-consequences analysis (CCA) to provide a comparative assessment of the costs and benefits of MBT compared to TAU. We will measure children's health and social care use on an adapted version of the Child and Adolescent Service Use Schedule (CA-SUS; Byford et al., 2007) and using a Service Use Record completed by CAMHS to record data on services offered and attended in CAMHS during the study period.

<u>Background and rationale</u>

In 2020, one in six (16.0%) children in England aged 5 to 16 were identified as having a probable mental disorder (Vizard et al., 2020). The majority of these children, when presenting to child mental health services, do not receive an evidence-based treatment. This is partly because most empirically tested treatments were developed to treat single disorders (Bearman & Weisz, 2015; Jepperson et al., 2021), whereas the evidence indicates that the structure of mental health symptoms does not straightforwardly map onto traditional diagnostic categories and comorbidity of mental health problems is high (McElroy et al., 2018; Rhee et al., 2015). Indeed, most children referred for treatment show high rates of transdiagnostic co-morbidity – this is the rule, not the exception (Garber & Weersing., 2010). As a result, there is a significant research-practice gap.

A solution to this research-practice gap is for clinical trials to focus on transdiagnostic interventions that target *causal and maintaining factors underlying a range of clinical presentations*. Transdiagnostic interventions are especially suitable for children, who show a great overlap in symptoms and a high level of transitory symptoms across developmental stages (Chu et al., 2016; Kennedy, 2018). Transdiagnostic interventions also have good potential for implementation, as they allow clinicians to address diverse diagnoses and sub-threshold symptoms within a single treatment model, and simultaneously target multiple problem areas, so limiting training burden on clinicians (Beauchaine & Cicchetti, 2019; Kennedy, 2018; Weissman et al., 2019).

Because of the ubiquity of emotion regulation (ER) difficulties across a wide range of psychopathologies, research has identified ER as a core transdiagnostic mechanism (Cludius et al., 2020). ER refers to implicit and explicit processes and strategies involved in regulating emotional states. Both adequate down-regulation of negative emotional states and up-regulation of positive states during childhood play a critical role in adaptive development and well-being (Daniel et al., 2020). ER difficulties have been associated with risk of psychopathology across both emotional and behavioural disorders (Compas et al., 2017; Kim-Spoon et al., 2013; Thomson et al., 2017). Findings

of a recent meta-analysis suggest that interventions can enhance ER in youth, and that these changes correlate with improvements in psychopathology (Moltrecht et al., 2021). However, of the 21 studies in the meta-analysis, only 4 targeted children aged 6-12, thereby highlighting a significant evidence gap for this age group. In addition, most research and interventions have neglected the role of positive/adaptive ER strategies, despite recent research highlighting their importance, especially for children and young people (Moltrecht et al., 2021).

The capacity to 'mentalize' is now recognised as a key component of positive or adaptive ER (Fonagy et al., 2002). Mentalizing has been defined as 'the process by which we make sense of each other and ourselves, implicitly and explicitly, in terms of subjective states and mental processes' (Bateman & Fonagy, 2010). The ability to mentalize one's own experiences and those of others plays a key role in coping with stress, regulation of emotions, and the formation of stable relationships (Fonagy et al., 2002). Empirical studies have shown that deficits in the capacity to mentalize are predictive of maladaptive ER in both clinical and non-clinical populations (Rothschild-Yakar et al., 2019; Schwarzer et al., 2021), and that there are deficits in mentalizing capacity among children with either internalizing or externalising psychopathology (Bizzi et al., 2019; Ensink et al., 2016; Halfon et al., 2017a; Halfon et al., 2020; Sharp et al., 2012).

The importance of parental mentalizing to the well-being of children is also clearly established. Parental mentalizing has been found to be significantly associated with both child's internalizing and externalizing symptoms and social–emotional competencies (Charpentier et al., 2022). The quality of a carer's mentalizing is positively associated with sensitive caregiving, strengthened parent-child relationships, and secure attachment (Huth-Bocks et al., 2014; Rutherford et al., 2015). Parents with higher mentalizing capacity are better able to tolerate emotional distress in their children, which is thought to be helpful in managing parenting stress (Rutherford et al., 2013). A systematic review exploring the association between parental mentalizing and children's mental health outcomes (Camoirano, 2017) highlighted that poor maternal mentalization is associated with overcontrolling parenting (Borelli et al., 2017),a higher incidence of child anxiety (Esbjørn et al., 2013), child emotion regulation difficulties (Heron-Delaney et al., 2016) and greater child externalising behaviours (Smaling et al., 2017).

Mentalization Based Treatment (MBT) was originally developed as a treatment for adults with a diagnosis of borderline personality disorder (Bateman & Fonagy, 2010), where emotion dysregulation is a key feature. The evidence base for its effectiveness with this group is good (Vogt & Norman, 2019). More recently it has been adapted for therapeutic work with children and families (Midgley & Vouvra, 2012). For school age children, there is preliminary evidence that promoting a child's mentalizing capacity can improve ER and symptomatic improvement, including children's behavioural and emotional difficulties (Halfon et al, 2017; Keaveny et al., 2012) Moreover, improvement in parents' capacity for mentalizing has been associated with reductions in children's internalizing and externalizing problems (Halfon & Besiroglu, 2021). However, as a recent systematic review has highlighted, high quality clinical trials of MBT for school-age children are lacking (Midgley et al., 2021), and time-limited MBT has yet to be evaluated as a transdiagnostic intervention for school-age children.

In summary, given that many children presenting to mental health services experience comorbid difficulties, there is a need for evidence-based transdiagnostic interventions that effectively target mechanisms underlying co-occurring mental health difficulties; ER is the best-evidenced mechanism implicated in a range of common mental health disorders. MBT is a well-evidenced therapy for a range of populations that aims to promote mentalization, which in turn increases ER capacities, leading to decreased emotional and behavioural difficulties. Until now, children under 12 have not had the benefit of this transdiagnostic approach, as the evidence for its effectiveness in this population has not yet been examined. The aim of this study is to test the clinical- and cost-effectiveness of MBT for school-age children with comorbid internalizing and externalizing difficulties. We will conduct the first randomised controlled trial of MBT for this population and examine the role of ER as a mediator of treatment response. If effective, we hope this scalable, transdiagnostic approach can become available to the growing number of children presenting to mental health services with a mix of internalizing and externalizing difficulties. In addition, it may enable a more efficient allocation of health and social care resources by reducing the need for longer-term, more intensive specialist mental health interventions.

Methods, materials and research plan

Trial design

This study will be a pragmatic, individually randomized, superiority trial comparing MBT with treatment as usual (TAU), with those administering outcome measures and analysing the data, blind to group assignment. An internal pilot will inform the optimal delivery of the main study, which will be a fully powered, randomised control trial of the intervention. Both the internal pilot and main trial will have an embedded qualitative component. Assessments of capacity for ER will enable mediator analyses to identify mechanisms underlying symptom change.

The internal pilot will take place before the main RCT, with six CAMHS services across two NHS Trusts,, following the same recruitment and data collection procedures as the main trial. The internal pilot will be an opportunity to test a) the process and procedures of training and supervising the CAMHS practitioners delivering MBT; b) the recruitment processes (including screening of referrals and contact with families); and c) the feasibility of data collection processes. With regard to this third aim, this will include feasibility of remotely conducting two task-based assessments of emotion regulation. Recent recommendations for the assessment of emotion regulation processes suggest that researchers adopt a multi-modal approach, where self-report measures are complemented with task-based and/or observational measures. Only few studies have done this sufficiently especially as part of clinical trials. (Moltrecht et al., 2021). Hence, one of our objectives for the internal pilot phase is to explore the feasibility of our multi-modal assessment approach as part of a clinical trial and test the convergence validity of the different ER measures. If successful, we will carry forward self-report and one or both task-based ER measures to the main trial where they may be employed at baseline only.

Research Questions and hypotheses

This study aims to evaluate the clinical- and cost-effectiveness of MBT in improving mental health outcomes for children aged 6-12 with mixed (internalizing and externalizing) mental health problems.

Hypothesis 1: Children allocated to MBT will experience a significantly greater reduction in mental health problems – both internalizing and externalizing – when compared with the TAU group.

Hypothesis 2: Families allocated to MBT will also experience a greater improvement in a range of secondary outcomes, including improved capacity for emotion regulation, and decreased parental stress, and a reduction in health and social service use and costs compared to the TAU group.

Hypothesis 3: The impact of treatment on child mental health will be mediated, in part, by changes in capacity for emotion regulation (in both parent and child).

The Implementation and Process Evaluation (IPE) aims to investigate: a) model fidelity, b) the experience of MBT (including the change process) from the perspective of service users; and c) any barriers to implementation and scalability post-trial.

Study setting

The study will be set in two large mental health trusts in the UK: Barnet, Enfield and Haringey (BEH) Mental Health NHS Trust and Oxford Health NHS Trust, which between them run 12 child and adolescent mental health services (CAMHS). Involvement with the study is supported at senior management level within these CAMHS. These services receive over 3,000 referrals of children aged 6-12 per year, from a diverse cultural and social background. The services are multi-disciplinary, including clinical psychologists, child psychiatrists, child psychotherapists, family therapists, social workers, primary mental health workers and clinical nurses.

Participants

320 children aged 6-12 with mixed (internalizing and externalizing) mental health problems, and their parents/carers. Children will be recruited from referrals to participating CAMH services.

Based on data available from services, approximately 60% of referrals are accepted as suitable for CAMHS, and a case file review has indicated that approximately 40% of accepted cases are likely to meet our inclusion criteria, with the majority of other referrals in this age group related to Autistic Spectrum Disorder (ASD) or neurodevelopmental disorders. This would make an estimated eligible pool of 720 children per year. We anticipate a consent rate of approximately 40-50%. The number of children included in each participating CAMHS team will be monitored by the trial team throughout the recruitment phase of the study. If the intake of children falls behind recruitment targets, appropriate action will be taken, including the possibility of adding additional clinical sites to the study.

During the internal pilot we will aim to recruit 40 families to the study (20 to MBT, 20 to Treatment as Usual). The research team will monitor recruitment during the pilot phase in order to identify any barriers to recruitment and to allow for any changes in procedure.

Eligibility criteria

Inclusion Criteria:

- Child aged 6-12 at time of randomisation;
- Carer-reported Strengths and Difficulties Questionnaire (SDQ):
    - total difficulties score for child of ≥14
    - emotional problems ≥5
    - conduct score ≥3
    - functional impairment score of ≥1.
- Valid informed consent

Exclusion criteria:

- Current participation in another mental health intervention trial. This includes where the family has previously participated in the ERiC study.
- Indications of, or pre-existing clinical diagnosis (in child or parent) of:
    - psychotic disorder
    - ASD
    - pervasive developmental disorder
    - eating disorder
    - severe learning difficulty
    - (in parent) severe substance abuse disorder.
- Children will also be excluded where the referring clinician identifies an immediate risk of harm to self or others.

Recruitment, screening and gaining informed consent

The study will recruit participants over a 26-month period (4 months for the internal pilot, and 14 months for the main trial, with the pilot sites continuing to recruit while data from the internal pilot is analysed and sites are set up for the main trial). Participants' journey through the trial is mapped out in Figure 1, and specific aspects are explained in more detail below.

Figure 1

CONSORT Diagram

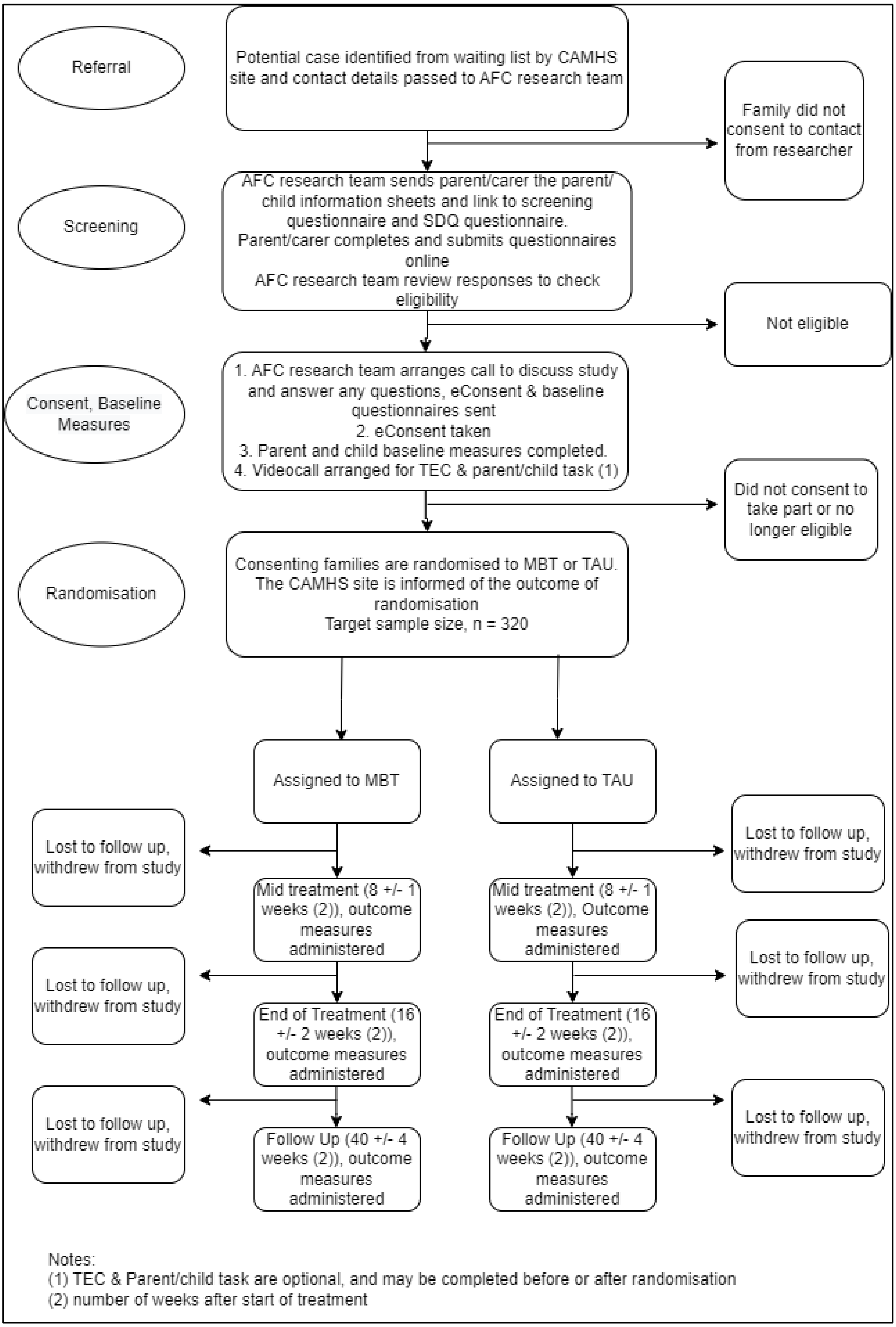

Initial identification and referral
Referrals on the CAMHS waiting list will be initially screened by a member of the CAMHS team to see if the child is expected to be aged 6-12 at the time of randomisation and ensure that there are no indications that any exclusion criteria apply. The parents/carers of those children who may be eligible will be offered further information about the study; verbal consent will be sought from the family to pass the parent's contact details to the Anna Freud Centre (AFC) research team. The call, and granting of verbal consent must be documented in the child's CAMHS patient record.

Screening
Following receipt of the online referral form, the AFC research team will send parent and child participant information sheets (PIS) to the parent/carer. The parent/carer will also be sent links to a screening questionnaire and SDQ questionnaire. The questionnaires will include eConsent for screening.

Consent
If, based on the information in the screening questionnaires, and the information provided on the site referral form, the child is shown to be eligible for the study, the AFC research team will contact the parent/carer to make an appointment with the parent/carer for a telephone/video call to discuss the study further and answer any questions the parent/carer may have.

The AFC research team will send the parent/carer a link to the eConsent form. eConsent can be given during/after this call, or, if the parent/carer requests more time to consider participation in the study, a further call will be arranged to answer any additional questions. eConsent must be given before the baseline measures are collected. Families can request to have a copy of the completed consent form emailed to them.

Children will be invited to provide eAssent to participate at study entry, and verbal assent will be sought by researchers prior to commencing interviews and tasks.

Baseline data collection
Mandatory elements; measures which must be completed prior to randomisation
Once informed consent has been granted, the parent/carer will be able to access and complete the parent and child baseline questionnaires and asked to complete them online. At the family's request, baseline measures can also be completed on a call with AFC staff.

Test of Emotional Comprehension and parent-child interaction task (optional element for consenting families only)
A video call with the family will be arranged for completion of the Test of Emotional Comprehension (TEC) task and parent-child interaction task. Wherever possible, this will be performed prior to randomisation. However, if this is not feasible, randomisation may proceed and the AFC research team will endeavour to arrange the call shortly after randomisation.

Where families have not consented to this part of the study, this video call will not be performed.

Randomisation

Once mandatory elements of baseline data collection have been completed, participants will be randomised to either MBT or TAU, stratified by CAMH service and age group (6-9 years and 10-12 years old), using permuted blocks of size 2 within each stratum. The randomisation sequence will be generated from random numbers generated in the R software for statistical computing (R Core Team, 2021) and pre-loaded into the REDCap data management platform, which will be used for data collection.

The randomisation sequence will be stored but concealed within the REDCap data capture system. When all information relevant for stratification (CAMH service and age) has been completed, a researcher has the option to initialise randomisation. This will allow the allocation to be revealed within the REDCap system.

The AFC research team will then email the CAMH service to inform them of the result of the randomisation, so that treatment can be initiated. The site staff must:

- Document the outcome of randomisation in the child's medical notes
- Inform the child's GP that the child is taking part in the ERiC trial what their allocated treatment is
- Complete a brief online form informing the AFC research team of the planned treatment start date, to allow for scheduling of subsequent assessments.

After randomisation, a certificate of participation will be provided to each family taking part in the study.

Interventions

Interventions in both arms of the study will be delivered by therapists working in the CAMHS teams taking part in the study. As part of study set up, all therapists taking part in the study will be randomly allocated to either deliver MBT or treatment as usual. Therapists will be stratified by NHS banding, which takes into consideration profession and level of experience, to ensure that any systematic differences between groups are due to the model of intervention. Therapists who have had previous training in MBT will by default be allocated to the MBT arm of the study.

MBT: The adaptation of MBT used in this study is a manualised, transdiagnostic model designed for children aged 6-12 and their carers. It aims to promote adaptive ER by attending to the capacity to understand oneself and others in terms of underlying mental states. MBT consists of 6-8 sessions, delivered fortnightly, which can flexibly involve different members of the family, with a primary focus on promoting mentalizing and emotion regulation in the parent-child relationship (see Figure 2). Therapists randomly allocated to deliver treatment for the intervention group will receive a 3-day MBT training, plus fortnightly group supervision to support treatment fidelity. Practitioners are supported by an interactive online treatment manual and the use of a self-completed MBT (child) Fidelity Scale (https://manuals.annafreud.org/mbt-c/index.html).

Figure 2
MBT Logic Model

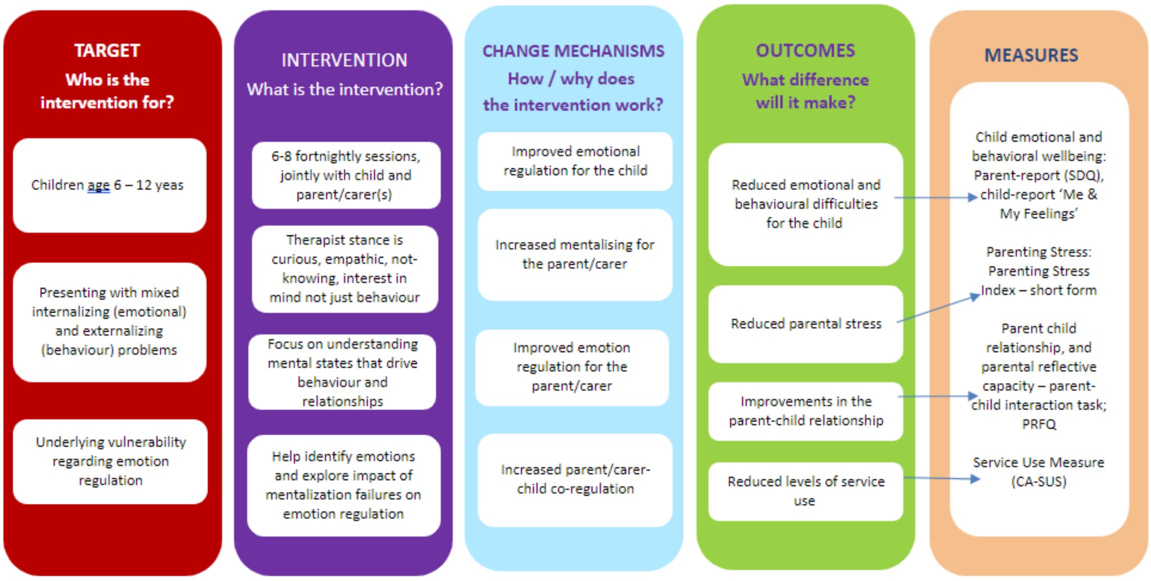

Treatment as Usual (TAU): The control group will be offered TAU. As there is no single evidence-based treatment for this group, and because practice is not standardised across child and adolescent mental health services (CAMHS) in England, TAU is likely to include CBT, parenting groups, and/or children's social skills groups. Scoping work with CAMH services has confirmed that 6-8 fortnightly sessions would be the usual length of intervention for this client group. Therapists will be provided with usual supervision, in line with existing practice in participating CAMHS teams. As part of the

study, more detailed data about TAU will be collected, including type of intervention, treatment intensity and professionals offering treatment**.**

Discontinuation and withdrawal
Children and families can withdraw from either treatment or the trial without further explanation at any point. Where children or families withdraw from treatment, they will be invited to continue to participate in the trial. Reasons for discontinuing or withdrawal from treatment or the trial will be recorded, where families agree to provide such a reason. Study treatment may be stopped early if this is felt by the CAMHS team to be in the child/family's best interests, but the family will remain part of the study for the purposes of follow up, unless the parent/carer explicitly withdraws consent for further involvement.

Data collection
There will be three types of data collected: questionnaires, online tasks (subject to testing of feasibility during the internal pilot phase) and a semi-structured interview.

Questionnaire data will be collected using a secure, browser-based web application for developing, maintaining, and managing different types of surveys and securing online/offline data collection (REDCap). Wherever possible, participants will self-complete these questionnaires by following a link to the REDCap webpage, however, there will also be an option to complete them via video or telephone call with a member of the research team. If collected during the video call, the responses to questionnaires will not be video recorded. One questionnaire (Service Use Record) will be completed by the participant's local CAMHS team.

There are two tasks: a parent-child interaction task, and a child task (the Test of Emotional Comprehension; TEC). These interactive tasks will take place during a video call between the family and a member of the research team and part of the video call will be recorded. The child's responses to the questions in the TEC task will be collected in real-time during the video call and entered directly into REDCap by the member of the research team conducting the call. The parent-child interaction task is facilitated by the member of the research team and the video call recorded; the recording is saved securely and later analysed and scored, and scores entered into REDCap by a member of the research team.

The semi-structured interviews are part of the implementation and process evaluation (see Outcome Measures). They will take place after treatment has ended during an online call and audio recorded by the research team. The recording will be saved securely at AFC and transcribed and analysed.

For families who do not have access to the technology needed to facilitate a video call, data collection will take place over a telephone call, or else families will be given the opportunity to access the necessary technology at the CAMHS where treatment is taking place. In order to ensure that recruitment is inclusive, interpreters will be made use of to support data collection, where required.

The use of REDCap automatically scores and transmits the data, reducing the risk of data entry error. All research assistants are trained on the administering of the outcome measures. A summary of data collection timepoints is provided in the schedule of enrolment, interventions, and assessments (SPIRIT schedule) below (Figure 3.)

Participants' personal data will be securely stored and only accessible by members of the ERiC research team at AFC. At the end of the study, data will be archived in a safe and secure location.

Blinding

Parent/carers and children in the trial will not be blinded to intervention group. However, research assistants and those involved in data collection and analysis (including coding of ER tasks) will be blind to intervention group.

Outcome measures

Brief, well-validated tasks and questionnaires will be administered at four points: baseline (pre-randomisation), 8 ±1 weeks after start of treatment (mid-treatment), 16 ±2 weeks after start of treatment (end of treatment) and 40 ±4 weeks after start of treatment (follow-up). An overview of all assessment points and outcomes are detailed in the SPIRIT (Figure 3)

In order to maximise clinical validity, data will be gathered through multiple methods (including questionnaire, interview and observational assessments) and from multiple perspectives (including self- and carer-reports). A consensus-based standard set of international measures (Krause et al., 2021) has been used. All questionnaire measures selected have established reliability, validity, utility/practicability and acceptability for this age group; they have also been reviewed by our parent co-applicants to ensure that they are acceptable, relevant and culturally appropriate. The pilot phase of the study will be used to test the feasibility of online, task-based assessment of parent-child ER capacity and mentalizing.

<u>Primary outcome measure:</u> Parent-rated Strengths and Difficulties Questionnaire (SDQ) Total Difficulties Score (Goodman, 2001). The SDQ is one of the most widely used mental health measures for children in the UK, covering both emotional (anxiety and depression) and behavioural (ADHD and conduct) symptoms.

<u>Secondary outcome measures</u> will include a range of carer- and child-report assessments and tasks to assess:

1) Emotional and behavioural problems,
   - Me and My Feelings (Deighton et al., 2013; Patalay et al., 2014) – a brief 16-item self-report measure for children, including both emotional and behavioural subscales

2) Personalized treatment goals,
   - Goal Based Outcomes (parent/carer-defined) (Law & Jacob, 2015) a personalized goal-based measure completed by parent/carers

3) Parenting stress,
   - Parental Stress Index – Short Form (Abidin, 2012; Haskett et al., 2006) a 36-item parent/carer-report measure of stress and the parent-child relationship;

4) Parental mentalizing capacity,
   - Parental Reflective Functioning Questionnaire (Luyten et al., 2017) an 18-item parent/carer-report measure of parental mentalizing;

5) Service use and costs.
   - Child and Adolescent Service Use Schedule (CA-SUS; Byford et al., 2007) - a questionnaire which records service use 3 months prior to baseline, during the course of the treatment, and between end of treatment and follow-up
   - Service Use Record- a questionnaire completed by CAMHS team to record the services offered and attended between baseline and end of treatment, and end of treatment and follow up.

6) Emotion regulation in child and parent will be assessed using the following:
   - Emotional Awareness Questionnaire (Rieffe et al., 2008) – a 30-item child self-report measure of emotional awareness and mentalizing capacity.
   - Test of Emotional Comprehension (Pons & Harris, 2000) – online task to assess child's capacity for emotional understanding, ER and mentalizing capacity. This task takes approximately 15 minutes to complete. The test is divided into a set of stories in an established order. The test booklet consists of illustrations with a story that is read for each situation. On every page, there are four possible outcomes represented by emotional facial expressions. Throughout the test, there are five options to choose from: happy, sad, angry, afraid, alright. The children are asked to assign an emotion represented by a facial

expression to the situation. A digitalised version of the test booklet will be used in the current study. Feasibility of its use will be tested in the internal pilot study.
- Emotion Regulation Checklist for Children (Shields & Cicchetti, 1997) - a 24-item parent/carer-report measure of child's ER capacity.
- Difficulties in Emotion Regulation Scale (Gratz & Roemer, 2004) – a 36-item parent/carer report measure of parent/carer's ER capacity.
- Parent-Child Interaction Task – a parent/carer-child discussion task to assess capacity of parent/carer to support ER and mentalizing in the parent/carer-child relationship, and to assess child's use of ER strategies. The researcher asks the child to recall a time when they felt sad, and to talk to the parent/carer about what happened and how it made them feel. After discussion, the interviewer asks the child: "What did you do to make your sad feelings go away?" followed by one additional prompt to elicit further details e.g., "What else did you do?". This is repeated for three other emotions: happy, afraid, angry. The task is video and audio recorded. The task is adapted from Davis et al. (2010) and Shipman and Zeman (1999) and takes approximately 15 minutes to complete; the feasibility of its use will be tested in the internal pilot study. The coding scheme developed by Davis et al. (2010) will be used to identify the child's use of emotion regulation strategies. An adapted version of the coding scheme developed by Vanwoerden (2020) will be used to assesses degree of positive mentalizing, and degree of negative or maladaptive mentalizing (hyper or hypo mentalizing) within the dyad.

7) Treatment fidelity
- Treatment fidelity will be assessed using the supervisor-report version of the MBT (Child) Fidelity Scale (Malberg et al., 2019), which can also be used to secondarily examine how specific elements of the MBT intervention relate to treatment outcome.

8) Implementation and Process Evaluation:
- The Experiences of Therapy and Research Interview (Midgley et al., 2011), a semi-structured interview carried out with parents and young people?, exploring the experience of change, the therapy process and of participating in research.
- Online survey – examining stakeholder views on the scalability of the MBT intervention at the end of the delivery phase.

Figure 3

SPIRIT Schedule

	**Study Period**				
	**Screening**	**Baseline**	**Mid treatment (8 ± 1 weeks[1])**	**End of treatment (16 ± 2 weeks[1])**	**Follow up (40 ± 4 weeks[1])**
**TIMEPOINT**	***-t₁***	***t₀***	***t₁***	***t₂***	***t₃***
**ENROLMENT:**					
**Eligibility screen**	X				
**Informed consent[2]**	X	X			
**Demographics**		X			

**Randomisation**		X			
**INTERVENTIONS:**					
MBT					
TAU					
**ASSESSMENTS[3]:**					
SDQ	X	X	X	X	X
Me and My Feelings, GBOs, PSI, PRFQ, ERC, DERS, EAQ		X	X	X	X
TEC, Parent-child interaction task*		X		X	
Experiences of therapy and research interview[4]				X	
CA-SUS		X		X	X
Service Use Record					X

Notes.

1. Weeks from start of treatment
2. The PIS will be sent prior to the screening assessment. Once the screening assessment is complete, a call will be arranged with the parent/carer to discuss the study. Consent may be given during or after this call, but if the parent/carer asks for more time, a further call can be scheduled. Consent must be given prior to commencement of baseline data collection.
3. Measures marked with a * will be tested for feasibility during the internal pilot, and carried forward into the main trial if feasible
4. The experiences of therapy and research interview will be conducted with consenting parents/carers subject to available resource. We aim to complete the interview with ≥20% of parents/carers.

See abbreviations list for full names of measures.

Monitoring of potential harm

To date there is a lack of agreement or guidelines on suitable approaches to assessing and monitoring potential harm in the context of clinical trials for psychological treatments (Jonsson et al., 2014), especially in the context of child and family treatments. For the purposes of this study, we will adopt a broad definition of a Serious Adverse Event (SAE). These include (in child or carer):

- Violent behaviour resulting in serious physical harm to another person
- Family relationship breakdown
- New or escalated self-harm
- New or escalated suicidal ideation (a preoccupation with suicide/thoughts about suicide, with no clear plans to take own life)

- Suicidal intent (concrete and deliberate plans to end own life, with a conscious desire to escape from the world and a resolve to act purposively in this regard, e.g., a suicide attempt. This may be a deliberate action or disclosing of a deliberate action)
- Other life-threatening events
- Hospitalisation due to drugs or alcohol, self-harm, or for psychiatric reasons, or unplanned in-patient hospitalisation for medical reasons
- Event resulting in significant disability/incapacity
- Death, including suicide

SAEs may occur during the course of being involved in the trial. This could be *irrespective of* intervention or data collection or may be *related to the* intervention or data collection. Ascertaining a relationship between an SAE and the intervention in the trial must be assessed on a case-by-case basis.

SAEs occurring between randomisation and 30 days post last trial therapy session must be reported using the SAE report form within 48 hours of becoming aware. If a serious event occurs more than 30 days post therapy that is considered directly related to the trial treatment, an SAE report form should also be submitted. As part of this process, the local lead investigator will be required to undertake an assessment of causal relationship to study treatment.

The Chief Investigator or appropriate delegate will conduct the Sponsor's causality of causal relationship to both study treatment and to study assessments. There is no reference safety information available for the study treatment, therefore all SAEs will be regarded as unexpected. SAEs which are both related and unexpected will be reported to the Research Ethics Committee (REC).

The Data Monitoring and Ethics Committee (DMEC) will regularly review SAE data arising from the study, and any concerns will be reported, along with the DMEC's recommendations, to the AFC study team and to the Trial Steering Committee (TSC), who must ratify any recommendations prior to implementation.

Internal pilot

During the internal pilot, 40 children and families will be recruited in a sub-sample of CAMHS teams. During this phase any potential barriers to training, recruitment, intervention delivery or data collection will be identified. Towards the end of the delivery phase, a problem-solving workshop will be held in each site included in the pilot phase of the trial, attended by trial therapists, service managers and any other relevant CAMHS staff to gain feedback on training, recruitment, delivery of intervention, and supervision procedures. Any issues arising, as well as potential adaptations, will be discussed in meetings of the Trial Management Group and the TSC, both during and upon completion of the internal pilot. Any recommended changes will be implemented for the full trial. The MBT training team will also review levels of model fidelity during the pilot phase, including any items on the scale which had the lowest mean ratings, and any adaptations to training and/or supervision that should be made will be identified, to improve model fidelity in the main study.

The internal pilot will also be an opportunity to examine alternative approaches to the assessment of emotion regulation. Although multiple measures exist to assess ER in children, most clinical trials do not include the perspectives of both parents/carers and children. Moreover, recent research has highlighted the importance of assessing different components of ER if we are to understand how ER relates to psychopathology, and which aspects of ER need to be targeted in interventions. Due to the primarily deficit-focused approach in clinical psychology, the field has long neglected the role of adaptive ER, especially in youth populations. This is of particular importance, because recent evidence shows that the lack of adaptive – but not presence of maladaptive - ER was significantly associated with psychopathology for children with emotional disorders (Braet et al., 2014). The pilot phase of this trial will therefore test the feasibility of evaluating different elements of ER, including self- and carer-reported assessment alongside task- and observation-based approaches. This will allow us to explore how different ER components relate to different symptom clusters and examine which aspects of ER change due to MBT treatment. If task- and observation-based approaches are shown

to be feasible, and there is indication that they add an additional perspective to the assessment of ER, including co-regulation in the parent-child relationship, they will be included in the full trial.

Data collected in the internal pilot will be used in the analysis for the full RCT: participants recruited in the pilot will be included within the total number of participants for the full trial.

Statistical plan and data analysis for main trial

Sample size and power calculation

The total sample size for the study will be 320. The power analysis was informed by an analysis of 71,763 UK CAMHS clinical records held by the Child Outcomes Research Consortium (CORC, https://www.corc.uk.net/), as well as by published psychotherapy trials in CAMHS settings. CORC data suggests that children meeting our inclusion criteria have a mean SDQ Total Difficulties score of 24.6 at presentation to CAMHS, with a standard deviation of 5.0. This score is our primary outcome. We assumed that the smallest clinically meaningful post-treatment difference on the SDQ is 1.5 points, which is equivalent to a standardised treatment effect difference of 0.3. The power analysis aimed to establish the number of children per therapist to give at least 80% power to detect this effect at the 5% significance level using a two-sided test. The statistical model will account for clustering of children within therapists, and of therapists within the CAMHS, via random intercepts. We will adjust for baseline SDQ scores.

We calculated the required sample size based on published formulae (Moerbek & Teerenstra, 2016) and made the following assumptions: The within-patient correlation was assumed to be $r_{within\text{-}patient} = 0.6$, which is close to the median value of 0.59 estimated by a meta-analysis of within-patient correlations (Balk et al., 2012). We estimated that 40 therapists will participate in the study and assumed a therapist effect of $ICC_{therapist} = 0.02$. We think this ICC is conservative. There is little data on therapist ICCs in CAMHS settings specifically, but the recent Improving Mood with Psychoanalytic and Cognitive Therapies (IMPACT) trial, set in English CAMHS, reported ICC < 0.01 (Goodyer et al., 2017). A smaller therapist ICC would increase power. The power analysis assumed that outcomes do not vary by CAMHS, which is also conservative. If there was intra-site correlation, power would increase, since each service would act as its own control. Given these assumptions, and allowing for 15% loss to follow-up, the total required sample size is $N = 320$ (160 children per treatment group, 8 children per therapist). The calculated power under this design is 80.8 %.

Statistical data analysis plan for the main trial

The primary analysis aims to establish the difference between the MBT and TAU groups in the SDQ Total Difficulties score at the end of treatment (16 weeks, Hypothesis 1). Children are clustered within therapists, who in turn are clustered within CAMH services. The primary analysis model is a linear mixed effects model, with random intercepts for therapists and services, controlling for SDQ and age group at baseline. The evidence for a difference in SDQ scores after treatment will be assessed via a two-sided t-test of the coefficient of the treatment indicator variable, using a parametric bootstrap to estimate standard errors. A 5% significance level will be used as a cut-off to support a finding of superiority of MBT over TAU.

Analogous models will be used to assess the evidence regarding a difference between the MBT and TAU groups with respect to secondary outcomes (Hypothesis 2).

To investigate potential mediation of the effect of MBT on child mental health via improvement in emotion regulation (Hypothesis 3), we will conduct a mediation analysis using treatment allocation as the exposure, the Emotion Awareness Questionnaire (EAQ) score as the mediator, and the SDQ Total Difficulties Score as the outcome. We will additionally adjust for both EAQ and SDQ scores at baseline. Summary scores from the validated scales will be used, rather than latent variable scores, in order to maintain consistency with the effect estimates of the primary analysis. We will conduct an analogous mediation analysis using parental emotion regulation, assessed by the Difficulties in Emotion Regulation Scale (DERS). More complex mediation models will be developed using exploratory analyses.

To examine whether the improvement in child SDQ Total Difficulties is clinically significant at the individual level, the reliable change index (Jacobson & Truax, 1991) will be calculated for each participant using the SDQ scores at baseline and post-intervention. Children will be classified as recovered, improved, unchanged, or deteriorated. We will compare the distribution of this classification in the MBT and TAU groups using an ordinal logistic regression model.

We will also estimate the difference in treatment effect between MBT and TAU on SDQ Total Difficulties and all secondary outcomes at 40-weak follow-up, using statistical models analogous to those for the primary analysis.

All primary and secondary analyses will be carried out as intention-to-treat analyses. If some values at the primary endpoint are missing, the following strategy will be employed: 1) Evaluate likely processes of missingness and assess their potential for causing bias; 2) Conduct a complete cases analysis as the primary analysis; (3) Conduct information-anchored sensitivity analyses using controlled multiple imputation under MNAR assumptions to gauge the sensitivity of the trial results to potential violations in the MAR/MCAR assumptions (Cro et al., 2016; 2019).

Economic Evaluation

The aim of the economic analysis will be to provide an assessment of the relative costs and benefits of MBT compared to TAU over the 40-week follow-up period. The study perspective will be that of the NHS and personal social services sectors, including those provided within the education sector. Health and social care service use will be collected using the CA-SUS questionnaire at baseline (covering the last 3 months) and at the 16- and 40-week follow-up points (covering the period since the last assessment). In addition, a Service Use Record will be completed by the CAMHS team at follow up to record the services offered and attended during the trial period. Collecting resource use at baseline will better characterise what ‘treatment as usual’ involves in terms of health care resources and allow for an adjustment for any potential baseline imbalances. Service use will be valued using PSSRU unit costs of health and social care (Jones & Burns, 2021) and national tariffs (NHS Reference Costs). MBT and TAU sessions will be costed using a bottom-up approach and using data on staff use (contacts). We will adopt a cost-consequences analysis (CCA) to report value for money of MBT versus TAU at 40 weeks. The CCA will report a disaggregated summary of all the costs and benefits of MBT versus TAU, without attempting to combine them into a single measure such as a cost-effectiveness or cost-utility ratio. This allows decision makers to decide which costs and benefits are most relevant to their decision context, and whether the relevant benefits are worth any additional costs that might be incurred. We will report mean costs and consequences, together with 95% confidence intervals, by treatment groups at baseline, 16 and 40 weeks. The consequences of interest for the CCA will be: SDQ and a range of carer and child-reported measures: The range of costs to be reported: intervention, medication, primary care, hospital care, social care, school-based service use. Costs and consequences will not be discounted given the short follow-up period.

Implementation and Process Evaluation

<u>Treatment fidelity</u>: Fidelity to the MBT model will be assessed using self-completion of the MBT Fidelity Scale by practitioners, and by supervisors.

<u>Experience of therapy and the change process from the service user perspective:</u> In order to examine the experience of therapy and the change process parents/carers and children who have agreed to an optional interview in the consent form will be interviewed at the end of intervention (16 weeks), using an adapted version of the Experiences of Therapy and Research Interview (Midgley et al., 2011), to explore their experiences of therapy and the change process. We aim to interview at least 20% of parents/carers and children. The interviews will explore parents’/carers’ and children's experiences of the intervention offered, how acceptable they found it, and any facilitators or barriers they found in engaging with the programme. For those who stopped attending the MBT, or who had low attendance, there will be an exploration of reasons for stopping/non-attendance, and of potential barriers to participation. Interviews will be audio-recorded and transcribed. Interviews with children may include the use of drawings, to enable a richer understanding of their experiences of therapy.

Interviews with children and parents/carers will be transcribed, and the data analysed using Framework Analysis (Parkinson et al., 2016), a qualitative method which allows both *a priori* issues and emergent data-driven themes to guide the development of the analytic process. Drawings, where included, will be incorporated at two stages of the analysis, using a specific qualitative analysis method used previously by members of the research team (Nunez et al., 2021).

<u>Implementation and potential for scalability:</u> A link to an online survey will be emailed to all stakeholders in the CAMHS taking part (practitioners, administrative staff, and managers) towards the end of the intervention phase to gain their views on the scalability of the MBT intervention at the end

of the delivery phase of the full RCT. Once the online survey is completed, a single problem-solving workshop, including members of the research team, and selected practitioners, administrative staff, and managers, will be organised, in order to review the findings of the survey and discuss potential ways in which any identified barriers to post-trial implementation could be overcome, if the intervention is demonstrated to be effective.

Definition of end of trial

The end of trial will be when the final data item for the final patient is received (i.e., it is anticipated that this will be when the final patient completes the 40-week follow up visit). At this point the 'declaration of end of trial' form will be completed and submitted to the Ethics Committee.

The AFC will advise sites on the procedures for closing the trial at site. Once the end of trial has been declared, no more prospective patient data will be collected but sites must cooperate with any data queries regarding existing data to allow for analysis and publication of results.

Trial oversight

The study will be held in the Child Attachment and Psychological Therapies Research Unit (ChAPTRe), a partnership between the Anna Freud Centre and University College London (UCL). Midgley (CI) has extensive experience in running pilot, feasibility and full-scale RCTs in local authorities and child mental health services, and expertise in incorporating qualitative methods into clinical trials. The study brings together a project team with diverse experience and expertise spanning: research design & statistical analysis, including data analysis of largescale clinical trials (Martin, UCL); assessment of emotion regulation (Moltrecht, UCL/AFC); health economic evaluation (Gomes, UCL); online data systems and management (Edbrooke-Childs, UCL/AFC); clinical delivery (Carter, BEH NHS Trust, and Marks, Oxford Health) and training and clinical supervision of mental health professionals (Morris, AFC). In addition, Prof. Fonagy (AFC/UCL) offers extensive experience in clinical trials, implementation, and policy impact. An expert-by-experience (Coffman) will review all procedures and materials, Chair the Parent/Carer Advisory Group and help to problem solve operational issues.

Two trial Managers will oversee day-to-day management of the trial, with two research assistants. Clinical management will be provided by Leads in CAMHS. A Trial Management Committee will meet regularly to manage the day-to-day running of the trial (membership to include the CI, trial managers, research assistants, and site leads). A Trial Steering Committee (TSC) will meet twice a year to provide oversight to ensure that the project is conducted according to the UK's Research Governance Framework. A Data Monitoring and Ethics Committee (DMEC) will oversee progression of the data collection and undertake regular monitoring of the study data. Unlike the TSC, the DMEC will have access to unblinded, comparative data. DMEC meetings will take place annually once data collection has commenced, usually two weeks prior to a TSC meeting in order for them to report to the TSC. Interim DMEC reviews may also be carried out where required, for example if the AFC study team have concerns, or annual DMEC review identifies a potential issue requiring closer monitoring.

User involvement

A parent co-applicant and an Advisory Group of parents and young people with experiences of child mental health services will continue to inform study design and delivery, specifically: a) recruitment of research staff; b) selection of outcome measures; c) ensuring information sheets and consent forms are designed with consideration of the needs of families; d) designing data management systems that have the confidence of families (timing of assessments, settings, confidentiality); e) co-developing structured qualitative interviews; and f) dissemination to public.

Ethical considerations

Ethical approval will be sought from the NHS Health Research Authority and necessary local permissions, and information governance approvals, will be obtained before patient recruitment. The research will be conducted in line with relevant ethical guidelines, including the Declaration of Helsinki. Voluntary and fully informed eConsent will be obtained from parents/carers and informed

assent will be sought from children. Families will be given Information Sheets (parent and child versions) in age-appropriate language, co-developed in collaboration with the Young Champions group at the Anna Freud Centre. Families will have the right to withdraw from the study at any point, without needing to give a reason. Their decisions concerning participation will not in any way affect their access to the usual support and treatment in CAMHS. Data will be anonymised, and personal details stored securely in accordance with General Data Protection Regulations 2018 (GDPR). Safety and child protection concerns will be closely monitored in line with safeguarding procedures. Independent oversight committees will monitor the safety of participants and child protection concerns. Where safeguarding concerns are identified, the CI and local site lead will be immediately informed, and local safeguarding policies followed to address concerns. The CI/study team will also liaise with the Anna Freud Centre safeguarding oversight group (SOG) where appropriate. Any safeguarding issues arising from the study design will be reported to the Trial Steering Committee, ethics committee, and funder as necessary.

Trial registration

The trial will be registered with the ISRCTN registry (International Standard Randomised Controlled Trial Number).

Data protection

A data protection impact assessment has been completed and agreed by the Data Protection Officer at the Anna Freud Centre. The data privacy notice is published online at www.annafreud.org/eric. All participants will be informed via the study information sheet of how to access the data privacy notice, in line with GDPR regulations.

Dissemination

The findings of the study will be disseminated to the academic and scientific community via conference presentations and publication of findings in leading peer-review journals.

Working with an Advisory Group of young people and parents/carers, we will create and publish a video summary of the full trial results and an infographic which will be disseminated through our social media and Learning Network of almost 30,000 allied professionals, enabling children and families to be better informed about evidence-based treatment models, and to empower practitioners to learn about the study findings. We will also create briefing notes which will be shared with policy makers.

If MBT is shown to be clinically- and cost-effective, dissemination into practice will be facilitated by the Anna Freud Centre's track-record of developing and delivering training programmes to mental health practitioners, including the development of Child Wellbeing Practitioners (CWP), as well as providing guidance to healthcare managers and commissioners of services, and to national and international bodies responsible for developing clinical guidelines (e.g., NICE). Training materials are already translated into several languages, and a web-based MBT treatment manual allows for rapid dissemination of training. There are clear pathways for practitioners who are trained during the study to go on to become supervisors themselves, thus embedding ownership and sustainability into the study design.

The UK's long-term plan for the NHS promises that 100% of children and young people who need specialist care can access it within a decade, but there is a deficit in evidence-based models that can be delivered by a wide range of professionals to children with a broad range of presenting difficulties; if this study demonstrates that primary mental health workers can be trained to deliver effective MBT, then this approach has the potential to become central to meeting the government's ambitions for child mental health services.

Discussion/Conclusion

One of the main barriers to children receiving evidence-based interventions in CAMHS is the lack of research evaluating *transdiagnostic* treatments that target mechanisms underlying the mixed internalising and externalising difficulties experienced by many young people. Research is often organised around specific diagnoses, but these children do not fit easily within diagnostic frameworks, meaning that there is a lack of evidence-based interventions available for them.

This trial will address this research-practice gap. Developed in collaboration with experts by experience and service providers, this study will be the first full-scale, sufficiently powered clinical trial to evaluate MBT as an intervention for school-age children with mixed emotional and behavioural difficulties. By targeting an underlying mechanism (ER), which has been shown to play a role in a range of mental health difficulties, it has the potential to identify a brief, manualized treatment that may be effective for a significant proportion of school-aged children referred to mental health services. MBT can be delivered by a range of mental health professionals in a real-world setting, mapping on well to the transdiagnostic THRIVE model used to organise child mental health services across the UK and internationally (Wolpert, 2019).

Further, the qualitative component of this research (the Experiences of Therapy and Research Interview; Midgley et al., 2011) will seek to identify what makes MBT ‘therapeutic’ from the perspective of families. Interviews will explore young people and parent/carers’ understanding of what makes treatment effective, why, and the role of ER in this process. This will provide valuable insights, informing our understanding of which treatments work for whom, and under what conditions (Fonagy et al., 2015).

This study is innovative in the use of multiple measures to assess different components of ER, integrating the perspectives of the child and the parent/carer. This will allow us to explore how different ER components relate to different symptom clusters and examine which aspects of ER change due to MBT treatment. Furthermore, most research focuses on ER within the individual, but for school-age children ER happens mostly in relationship to others; by examining parent-child co-regulation of emotions, this study represents a significant methodological advancement. In this way, this study is aligned with the “Roadmap for Mental Health Research in Europe” (Haro et al., 2014), in that it seeks to identify and interrogate putative mechanisms of change.

Many studies evaluating treatments in child mental health settings are limited by a lack of follow-up (Pilling et al., 2020). This study is ambitious in that it will include a six-month follow-up post-intervention. If MBT proves to be superior to TAU in the first 40 weeks, we will ask families for consent to be re-contacted, so that a long-term follow-up study can answer the question of whether the beneficial effects are maintained two and five years from baseline.

The Medical Research Council guidance on evaluation of complex interventions (Shahsavari et al., 2020) emphasises the importance of including a process evaluation and an economic evaluation within clinical trials. This study is ambitious in including both. The health-economic evaluation will improve our understanding of the economic implications, and value for money, of implementing MBT compared to current treatment options. In doing so, the trial will better map and evaluate ‘treatment as usual’ for a group of children who are poorly served by current evidence-based treatment guidelines, as they do not fit easily within existing diagnostic frameworks. The process evaluation will help to explain discrepancies between expected and observed outcomes, to understand how context influences outcomes, and to provide insights to aid implementation. Taken together, the health-economic and process evaluation will ensure that the findings of this evaluation are relevant and actionable for decision-makers, ensuring real world impact for children and families.

It is well established that child mental health is strongly associated with levels of deprivation and poverty (Morrison Gutman et al., 2015) and the Covid-19 pandemic is likely to have further increased inequalities in mental health. The Centre for Mental Health predicts that up to 1.5 million children and young people in the UK aged under 18 will need new or additional mental health support as a direct consequence of the crisis (O’Shea, 2021). Developing effective interventions for these children and families may help to mitigate health inequalities across the lifespan, hopefully preventing difficulties from becoming entrenched and persisting into adulthood. In evaluating MBT for children aged 6-12, the project will address the UN Sustainable Development Goal: ‘ensure healthy lives and promote well-being for all at all ages.’

Abbreviations

AFC- Anna Freud Centre
ADHD- Attention Deficit Hyperactivity Disorder
ASD- Autism Spectrum Disorder
BEH- Barnet, Enfield, Haringey
CAMHS - child and adolescent mental health services
CBT - Cognitive Behavioural Therapy
CCA- Cost-consequences analysis
CONSORT- CONsolidated Standards Of Reporting Trials
CORC- Child Outcomes Research Consortium
GBOs – Goal Based Outcomes
CA-SUS – child and adolescent service use questionnaire
CCA- cost consequences analysis
ChAPTRe- Child Attachment and Psychological Therapies Research Unit
CWPs- Child Well-being Practitioners
DERS – Difficulties in Emotion Regulation Scale
DMEC- Data Monitoring and Ethics Committee
EAQ – Emotional Awareness Questionnaire
ER - emotion regulation
GDPR - General Data Protection Regulations
MAR- missing at random
MCAR- missing completely at random
MBT - mentalization based treatment
MNAR- missing not at random
NICE- National Institute for Health and Clinical Excellence
PRFQ – Parental Reflective Functioning Questionnaire
PSSRU- Personal Social Services Research Unit
RCT- Randomised Control Trial
REDCap- Research Electronic Data Capture
SAE- Serious Adverse Event
SDQ – Strengths and Difficulties Questionnaire
SOG- Safeguarding Oversight Group
SPIRIT- Standard Protocol Items: Recommendations for Interventional Trials
TAU - Treatment as usual
TEC – Test of Emotional Comprehension
TSC- Trial Steering Committee
UCL- University College London

References

Abidin, Richard. Parenting Stress Index. (2012). Odessa, FL: Psychological Assessment Resources.

Bateman, A., & Fonagy, P. (2010). Mentalization based treatment for borderline personality disorder. World Psychiatry 9(1), 11–15. https://doi.org/10.1002/j.2051-5545.2010.tb00255.x

Balk, E. M., Earley, A., Patel, K., Trikalinos, T. A., & Dahabreh, I. J. (2012). Empirical Assessment of Within-Arm Correlation Imputation in Trials of Continuous Outcomes. Methods Research Reports, 12(EHC141), EF. Retrieved from http://www.ncbi.nlm.nih.gov/pubmed/23326900

Bearman, S.K. and Weisz, J.R. (2015). Review: Comprehensive treatments for youth comorbidity – evidence-guided approaches to a complicated problem. Child Adolesc Ment Health, 20: 131-141. https://doi.org/10.1111/camh.12092

Beauchaine, T., & Cicchetti, D. (2019). Emotion dysregulation and emerging psychopathology: A transdiagnostic, transdisciplinary perspective. Development and Psychopathology, 31(3), 799-804. https://doi.org/10.1017/S0954579419000671

Bizzi, F., Ensink, K., Borelli, J. L., Mora, S. C., & Cavanna, D. (2019). Attachment and reflective functioning in children with somatic symptom disorders and disruptive behavior disorders. European child & adolescent psychiatry, 28(5), 705–717. https://doi.org/10.1007/s00787-018-1238-5

Borelli, J. L., Hong, K., Rasmussen, H. F., & Smiley, P. A. (2017). Reflective functioning, physiological reactivity, and overcontrol in mothers: Links with school-aged children's reflective functioning. Developmental psychology, 53(9), 1680–1693. https://doi.org/10.1037/dev0000371

Braet, C., Theuwis, L., Van Durme, K. *et al.* (2014) Emotion Regulation in Children with Emotional Problems. *Cogn Ther Res* **38,** 493–504. https://doi.org/10.1007/s10608-014-9616-x

Byford, S., Barrett, B., Roberts, C., Wilkinson, P., Dubicka, B., Kelvin, R., . . . Goodyer, I. (2007). “Cost-effectiveness of selective serotonin reuptake inhibitors and routine specialist care with and without cognitive behavioural therapy in adolescents with major depression.” The British Journal of Psychiatry191, 521-7. https://doi.org/10.1192/bjp.bp.107.038984

Bunford, N., Dawson, A. E., Evans, S. W., Ray, A. R., Langberg, J. M., Owens, J. S., DuPaul, G. J., & Allan, D. M. (2020). The Difficulties in Emotion Regulation Scale-Parent Report: A Psychometric Investigation Examining Adolescents With and Without ADHD. Assessment, 27(5), 921–940. https://doi.org/10.1177/1073191118792307

Camoirano A. (2017). Mentalizing Makes Parenting Work: A Review about Parental Reflective Functioning and Clinical Interventions to Improve It. Frontiers in psychology, 8, 14. https://doi.org/10.3389/fpsyg.2017.00014

Charpentier Mora, S.; Bastianoni, C.; Koren-Karie, N.; Cavanna, D.; Tironi, M.; Bizzi, F. (2022). Parental Mentalizing during Middle Childhood: How Is the Adoption of a Reflective Stance Associated with Child’s Psychological Outcomes? Int. J. Environ. Res. Public Health, 19, 6205. https://doi.org/10.3390/ijerph19106205

Chu, B. C., Crocco, S. T., Esseling, P., Areizaga, M. J., Lindner, A. M., & Skriner, L. C. (2016). Transdiagnostic group behavioral activation and exposure therapy for youth anxiety and depression: Initial randomized controlled trial. Behaviour Research and Therapy, 76, 65–75. https://doi.org/10.1016/j.brat.2015.11.005

Chu, B. C., Temkin, A. B., & Toffey, K. (2016) Transdiagnostic Mechanisms and Treatment for Children and Adolescents: An Emerging Field, Oxford handbooks online. https://doi.org/10.1093/oxfordhb/9780199935291.013.10

Cludius, B., Mennin, D., & Ehring, T. (2020). Emotion regulation as a transdiagnostic process. Emotion (Washington, D.C.), 20(1), 37–42. https://doi.org/10.1037/emo0000646

Compas, B. E., Jaser, S. S., Bettis, A. H., Watson, K. H., Gruhn, M. A., Dunbar, J. P., Williams, E., & Thigpen, J. C. (2017). Coping, emotion regulation, and psychopathology in childhood and adolescence: A meta-analysis and narrative review. Psychological bulletin, 143(9), 939–991. https://doi.org/10.1037/bul0000110

Cro, S., Carpenter, J.R., Kenward, M.G. (2019). Information-anchored sensitivity analysis: theory and application, The Authors Journal of the Royal Statistical Society: Series A (Statistics in Society).

Cro, S.M., Morris, T.P., Kenward, M.G., Carpenter, J.R. (2016). Reference-based sensitivity analysis via multiple imputation for longitudinal trials with protocol deviation, Stata Journal, Vol: 16, Pages: 443-463, ISSN: 1536-867X

Daniel, S.K., Abdel-Baki, R. & Hall, G.B. (2020) The Protective Effect of Emotion Regulation on Child and Adolescent Wellbeing. J Child Fam Stud 29, 2010–2027. https://doi.org/10.1007/s10826-020-01731-3

Davis, E. L., Levine, L. J., Lench, H. C., & Quas, J. A. (2010). Metacognitive emotion regulation: children's awareness that changing thoughts and goals can alleviate negative emotions. Emotion (Washington, D.C.), 10(4), 498–510. https://doi.org/10.1037/a0018428

Deighton, J., Tymms, P., Vostanis, P., Belsky, J., Fonagy, P., Brown, A., Martin, A., Patalay, P. & Wolpert, W. (2013). The Development of a School-Based Measure of Child Mental Health. Journal of Psychoeducational Assessment, 31:247. https://doi.org/10.1177/0734282912465570

Ensink, K., Bégin, M., Normandin, L., & Fonagy, P. (2016). Maternal and child reflective functioning in the context of child sexual abuse: pathways to depression and externalising difficulties. European Journal of Psychotraumatology, 7, 30611. https://doi.org/10.3402/ejpt.v7.30611

Esbjørn, B. H., Pedersen, S. H., Daniel, S. I., Hald, H. H., Holm, J. M., & Steele, H. (2013). Anxiety levels in clinically referred children and their parents: examining the unique influence of self-reported attachment styles and interview-based reflective functioning in mothers and fathers. The British journal of clinical psychology, 52(4), 394–407. https://doi.org/10.1111/bjc.12024

Fonagy, P., Gergely, G., Jurist, E., and Target, M. (2002). Affect Regulation, Mentalization, and the Development of the Self. New York, N.Y: Other Press.

Fonagy, P., Target, M., Cottrell, D., Phillips, J., and Kurtz, Z. (2015). What works for whom? A critical review of treatments for children and adolescents. New York: Guilford, pp. 640

Garber, J., & Weersing, V. R. (2010). Comorbidity of anxiety and depression in youth: Implications for treatment and prevention. Clinical Psychology: Science and Practice, 17(4), 293–306. https://doi.org/10.1111/j.1468-2850.2010.01221.x

Goodman. R. (2001). Psychometric properties of the strengths and difficulties questionnaire. Journal of the American Academy of Child and Adolescent Psychiatry, 40 (11), 1337-1345.

Goodyer, I. M., Reynolds, S., Barrett, B., Byford, S., Dubicka, B., Hill, J., ... & Fonagy, P. (2017). Cognitive behavioural therapy and short-term psychoanalytical psychotherapy versus a brief psychosocial intervention in adolescents with unipolar major depressive disorder (IMPACT): a multicentre, pragmatic, observer-blind, randomised controlled superiority trial. *The Lancet Psychiatry*, *4*(2), 109-119.

Gratz, K. L., and Roemer, L. (2004). Multidimensional assessment of emotion regulation and dysregulation: development, factor structure, and initial validation of the difficulties in emotion regulation scale. *J. Psychopathol. Behav. Assess.* 26, 41–54. doi: 10.1023/B:JOBA.0000007455.08539.94

Halfon, S., Bekar, O., & Gürleyen, B. (2017). An empirical analysis of mental state talk and affect regulation in two single-cases of psychodynamic child therapy. Psychotherapy (Chicago, Ill.), 54(2), 207–219. https://doi.org/10.1037/pst0000113

Halfon, S., Bekar, O., & Gürleyen, B. (2017a). An empirical analysis of mental state talk and affect regulation in two single-cases of psychodynamic child therapy. Psychotherapy, 54(2), 207–219. https://doi.org/10.1037/pst0000113

Halfon, S., Coşkun, A., Bekar, Ö., & Steele, H. (2020) Imbalances and Impairments in Mental State Talk of Children with Internalizing and Externalizing Problems, Journal of Infant, Child, and Adolescent Psychotherapy, 19:3, 283-302, DOI: 10.1080/15289168.2020.1771531

Halfon, S., & Besiroglu, B. (2021). Parental reflective function and children's attachment-based mental state talk as predictors of outcome in psychodynamic child psychotherapy. Psychotherapy, 58(1), 81–94. https://doi.org/10.1037/pst0000347

Haro, J.M., Ayso-Mateos, J.L., Bitter, I., Demotes-Mainard, J., Leboyer, M., Lewis, S.W., Linszen, D., Maj, M., McDaid, D., Meyer-Lindenberg, A., Robbins, T.W., Schumann, G., Thornicroft, G., van der Feltz-Cornelis, C., van Os J., Wahlbeck, K., Wittchen, H.-U., Wykes, T., Arango, C., Bickenbach, J., Brunn :, Cammarata, P., Chevreul, K., Evans-Lacko, S., Finocchiaro, C., Fiorillo, A., Forsman, A.K., Hazo, J.-B., Knappe, S., Kueppe,r R., Luciano M., Mire,t M., Obradors-Tarragó, C., Pagano, G., Papp, S., Walker-Tilley, T. (2014). "ROAMER: roadmap for mental health research in Europe." International Journal of Methods in Psychiatric Research 23,Suppl 1: 1-14.

Haskett, M. E., Ahern, L. S., Ward, C. S., & Allaire, J. C. (2006). Factor structure and validity of the parenting stress index-short form. Journal of clinical child and adolescent psychology: the official journal for the Society of Clinical Child and Adolescent Psychology, American Psychological Association, Division 53, 35(2), 302–312. https://doi.org/10.1207/s15374424jccp3502_14

Heron-Delaney, M., Kenardy, J.A., Brown, E.A., Jardine,C., Bogossian,F., Neuman, L. et al. (2016). Early maternal reflective functioning and infant emotional regulation in a preterm infant sample at 6 months corrected age. J. Pediatr.Psychol. 41, 906–914. https://doi.org/10.1093/jpepsy/jsv169

Huth-Bocks, A. C., Muzik, M., Beeghly, M., Earls, L., & Stacks, A. M. (2014). Secure base scripts are associated with maternal parenting behavior across contexts and reflective functioning among trauma-exposed mothers. Attachment & Human Development, 16(6), 535–556. https://doi.org/10.1080/14616734.2014.967787

Jacobson, N S, and P Truax. (1991). Clinical significance: a statistical approach to defining meaningful change in psychotherapy research. Journal of consulting and clinical psychology vol. 59(1), 12-9. https://doi.org/10.1037/0022-006X.59.1.12

Jeppesen, P., Wolf, R. T., Nielsen, S. M., Christensen, R., Plessen, K. J., Bilenberg, N., Thomsen, P. H., Thastum, M., Neumer, S. P., Puggaard, L. B., Agner Pedersen, M. M., Pagsberg, A. K., Silverman, W. K., & Correll, C. U. (2021). Effectiveness of Transdiagnostic Cognitive-Behavioral Psychotherapy Compared With Management as Usual for Youth With Common Mental Health Problems: A Randomized Clinical Trial. JAMA Psychiatry, 78(3), 250–260. https://doi.org/10.1001/jamapsychiatry.2020.4045

Johns, R. G., Barkham, M., Kellett, S., & Saxon, D. (2019). A systematic review of therapist effects: A critical narrative update and refinement to review. Clinical Psychology Review, 67, 78–93. https://doi.org/10.1016/j.cpr.2018.08.004

Jones, K. & Burns, A. (2021) Unit Costs of Health and Social Care 2021. Personal Social Services Research Unit, University of Kent, Canterbury. https://www.pssru.ac.uk/project-pages/unit-costs/unit-costs-2020/

Jonsson U, Alaie I, Parling T, Arnberg FK. (2014). Reporting of harms in randomized controlled trials of psychological interventions for mental and behavioral disorders: a review of current practice. Contemp Clin Trials 2014 May;38(1):1-8 https://doi.org/10.1016/j.cct.2014.02.00

Keaveny, E., Midgley, N., Asen, E., Bevington, D., Fearon, P., Fonagy, P., Jennings-Hobbs, R., & Wood, S. (2012). Minding the family mind: The development and initial evaluation of mentalization-based treatment for families. In N. Midgley & I. Vrouva (Eds.), Minding the child: Mentalization-based interventions with children, young people and their families (pp. 98–112). Routledge/Taylor & Francis Group.

Kennedy, S. M., Tonarely, N. A., Sherman, J. A., & Ehrenreich-May, J. (2018). Predictors of treatment outcome for the unified protocol for transdiagnostic treatment of emotional disorders in children (UP-C). Journal of Anxiety Disorders, 57, 66–75. https://doi.org/10.1016/j.janxdis.2018.05.004

Kim-Spoon, J., Cicchetti, D., & Rogosch, F. A. (2013). A longitudinal study of emotion regulation, emotion lability-negativity, and internalizing symptomatology in maltreated and nonmaltreated children. Child Development, 84(2), 512–527. https://doi.org/10.1111/j.1467-8624.2012.01857.x

Krause, K. R., Chung, S., Adewuya, A. O., Albano, A. M., Babins-Wagner, R., Birkinshaw, L., Brann, P., Creswell, C., Delaney, K., Falissard, B., Forrest, C. B., Hudson, J. L., Ishikawa, S. I., Khatwani, M., Kieling, C., Krause, J., Malik, K., Martínez, V., Mughal, F., Ollendick, T. H., … Wolpert, M. (2021). International consensus on a standard set of outcome measures for child and youth anxiety, depression, obsessive-compulsive disorder, and post-traumatic stress disorder. The Lancet Psychiatry, 8(1), 76–86. https://doi.org/10.1016/S2215-0366(20)30356-4

Law, D. & Jacob, J. (2015). Goals and Goal Based Outcomes (GBOs): Some useful information. Third Edition. London, UK: CAMHS Press.

Luyten P, Mayes LC, Nijssens L, Fonagy P. (2017). The parental reflective functioning questionnaire: Development and preliminary validation. PLOS ONE. 12(5):e0176218. doi: 10.1371/journal.pone.0176218

Malberg, N.; Bate, J.; Midgley, N. (2019). MBT-C Adherence and Competence Scale. Unpublished manual.

McElroy, E., Shevlin, M., Murphy, J. et al. (2018). Co-occurring internalizing and externalizing psychopathology in childhood and adolescence: a network approach. Eur Child Adolesc Psychiatry 27, 1449–1457 (2018). https://doi.org/10.1007/s00787-018-1128-x

Midgley, N., Ansaldo, F., Parkinson, S., Holmes, J., Stapley, E., & Target, M. (2011). Experience of therapy interview (Young person, parent and therapist versions) [Unpublished manuscript]. Anna Freud Centre.

Midgley, N. & Vrouva, I. (2012). Mentalization-based interventions with children, young people, and families. London: Routledge.

Midgley, N., Sprecher, E. A., & Sleed, M. (2021). Mentalization-Based Interventions for Children Aged 6-12 and Their Carers: A Narrative Systematic Review,. Journal of Infant, Child and Adolescent Psychotherapy, 20(2), 169-189, DOI: 10.1080/15289168.2021.1915654

Moerbek M, Teerenstra S. (2016). Power analysis of trials with multilevel data. Boca Raton, FL: CRC Press.

Moltrecht, B., Deighton, J., Patalay, P., & Edbrooke-Childs, J. (2021). Effectiveness of current psychological interventions to improve emotion regulation in youth: a meta-analysis. European Child & Adolescent Psychiatry, 30(6), 829–848. https://doi.org/10.1007/s00787-020-01498-4

Morrison Gutman, L., Joshi, H., Parsonage, M. & Schoon, I. (2015) Children of the new century: mental health findings from the Millenium Cohort Study. London: Centre for Mental Health.

Núñez, L., Midgley, N., Capella, C., Alamo, N., Mortimer, R., & Krause, M. (2021). The therapeutic relationship in child psychotherapy: integrating the perspectives of children, parents and therapists. Psychotherapy research : journal of the Society for Psychotherapy Research, 1–13. Advance online publication. https://doi.org/10.1080/10503307.2021.1876946

O'Shea, N. (2021). Covid-19 and the nation's mental health Forecasting needs and risks in the UK: May 2021.
https://www.centreformentalhealth.org.uk/sites/default/files/publication/download/CentreforMentalHealth_COVID_MH_Forecasting4_May21.pdf

Parkinson, S., Eatough, V., Holmes, J., Stapley, E. and Midgley, N. (2016). Framework Analysis: A worked example of a study exploring young people's experiences of depression, Qualitative Research in Psychology, 13(2): 109-129. https://doi.org/10.1080/14780887.2015.1119228

Patalay, P., Deighton, J., Fonagy, P., Vostanis, P., & Wolpert, M. (2014). Clinical validity of the Me and My School questionnaire: a self-report mental health measure for children and adolescents. Child and Adolescent Psychiatry and Mental Health, 8(17), https://doi.org/10.1186/1753-2000-8-17

Pilling, S., Fonagy, P., Allison, E., Barnett, P., Campbell, C., Constantinou, M., Gardner, T., Lorenzini, N., Matthews, H., Ryan, A., Sacchetti, S., Truscott, A., Ventura, T., Watchorn, K., Whittington, C., and Kendall, T. (2020) Long-term outcomes of psychological interventions on children and young people's mental health: A systematic review and meta-analysis. PLoS ONE 15(11): e0236525. https://doi.org/10.1371/journal.pone.0236525

Pons, Francisco. and Harris, Paul. TEC (Test of Emotion Comprehension) (Oxford, England: Oxford University Press, 2000)

R Core Team (2021). R: A language and environment for statistical computing. R Foundation for Statistical Computing, Vienna, Austria. https://www.R-project.org/

Rhee, S. H., Lahey, B. B., & Waldman, I. D. (2015). Comorbidity Among Dimensions of Childhood Psychopathology: Converging Evidence from Behavior Genetics. Child Development Perspectives, 9(1), 26–31. https://doi.org/10.1111/cdep.12102

Rieffe, C., Oosterveld, P., Miers, A.C., Meerum Terwogt, M., & Ly, V. (2008). Emotion awareness and internalising symptoms in children and adolescents; the Emotion Awareness Questionnaire revised. Personality and Individual Differences, 45, 756-761.

Rothschild-Yakar, L., Stein, D., Goshen, D., Shoval, G., Yacobi, A., Eger, G., Kartin, B., & Gur, E. (2019). Mentalizing Self and Other and Affect Regulation Patterns in Anorexia and Depression. Frontiers in Psychology, 10, 2223. https://doi.org/10.3389/fpsyg.2019.02223

Rutherford, H. J., Booth, C. R., Luyten, P., Bridgett, D. J., & Mayes, L. C. (2015). Investigating the association between parental reflective functioning and distress tolerance in motherhood. Infant Behavior & Development, 40, 54–63. https://doi.org/10.1016/j.infbeh.2015.04.005

Rutherford, H. J., Goldberg, B., Luyten, P., Bridgett, D. J., & Mayes, L. C. (2013). Parental reflective functioning is associated with tolerance of infant distress but not general distress: evidence for a specific relationship using a simulated baby paradigm. Infant Behavior & Development, 36(4), 635–641. https://doi.org/10.1016/j.infbeh.2013.06.008

Schwarzer, N., Nolte, T., Fonagy, P., & Gingelmaier, S. (2021) Mentalizing and emotion regulation: Evidence from a nonclinical sample, International Forum of Psychoanalysis, 30:1, 34-45, DOI: 10.1080/0803706X.2021.1873418

Shahsavari, H., Matourypour, P., Ghiyasvandian, S., Golestan Nejad, M.R. 2020) "Medical Research Council framework for development and evaluation of complex interventions: A comprehensive guidance." Journal of Education and Health Promotion vol. 9,88. 28 Apr. https://doi.org/10.4103/jehp.jehp_649_19

Sharp, Carla & Venta, Amanda (2012). Mentalizing problems in children and adolescents. In: Minding the child: Mentalization-based interventions with children, young people and their families Edited by: Nick Midgley & Ioanna Vrouva (Routledge/Taylor & Francis Group, 2012). pp. 35–53.

Shields, A., & Cicchetti, D. (1997). Emotion regulation among school-age children: the development and validation of a new criterion Q-sort scale. Developmental Psychology, 33(6), 906–916. https://doi.org/10.1037//0012-1649.33.6.906

Shipman, K. L., & Zeman, J. (1999). Emotional understanding: a comparison of physically maltreating and nonmaltreating mother-child dyads. Journal of Clinical Child Psychology, 28(3), 407–417. https://doi.org/10.1207/S15374424jccp280313

Smaling, H. J., Huijbregts, S. C., van der Heijden, K. B., Hay, D. F., van Goozen, S. H., & Swaab, H. (2017). Prenatal Reflective Functioning and Development of Aggression in Infancy: the Roles of Maternal Intrusiveness and Sensitivity. Journal of Abnormal Child Psychology, 45(2), 237–248. https://doi.org/10.1007/s10802-016-0177-1

Thomson, N., Centifanti, L., Lemerise, E. (2017) Emotion Regulation and Conduct Disorder: the role of Callous-Unemotional Traits. In Essau, C., LeBlanc, S., Ollendick, T. (Eds) Emotion Regulation and Psychopathology in Children and Adolescents. Oxford: Oxford University Press.

Vanwoerden, S. (2020) The Development and Validation of an Observational Coding System for Real-Time Parent-Adolescent Mentalizing. PhD Thesis, available at: https://hdl.handle.net/10657/7266

Vizard, T., Sadler, K., Ford, T., Newlove-Delgado, T., McManus, S., Marcheselli, F., Davis, J., Williams, T., Leach, C., Mandalia, D., Cartwright, C., Thandi, S. (2020) Mental Health of Children and Young People in England, 2020) Wave 1 follow up to the 2017 survey. Available at: https://digital.nhs.uk/data-and-information/publications/statistical/mental-health-of-children-and-young-people-in-england/2020-wave-1-follow-up/copyright

Vogt, K.S. & Norman, P. (2019). Is mentalization-based therapy effective in treating the symptoms of borderline personality disorder? A systematic review. Psychology and Psychotherapy, 19(4), 441-464 **https://doi.org/10.1111/papt.12194**

Weissman, D. G., Bitran, D., Miller, A. B., Schaefer, J. D., Sheridan, M. A., & McLaughlin, K. A. (2019). Difficulties with emotion regulation as a transdiagnostic mechanism linking child maltreatment with the emergence of psychopathology. Development and Psychopathology, *31*(3), 899-915. https://doi.org/10.1017/S0954579419000348

Wolpert, M. (2019). THRIVE Framework for system change. London: CAMHS Press.
