# Supplementary material for: Emotion regulation in children (ERiC): A protocol for a randomised clinical trial to evaluate the clinical and cost effectiveness of Mentalization Based Treatment (MBT) vs Treatment as Usual for school-age children with mixed emotional and behavioural difficulties
Source: PLoS One. 2023 Aug 17;18(8):e0289503. doi: 10.1371/journal.pone.0289503 (PMC10434917; doi:10.1371/journal.pone.0289503)
Supplement: S2 File — (PDF) [file pone.0289503.s003.pdf]

IRAS ID: 316392

Participant Identification Number:

CONSENT FORM for PARENT/CARER

Title of Project: **Emotion Regulation in Children (ERiC)**

		I agree	I do not agree (for optional elements)
1	I confirm that I have read the information sheet version xxx dated xxx for the above study. I have had the opportunity to consider the information, ask questions and have had these answered satisfactorily.	☐	
2	I understand that my participation is voluntary and that I am free to withdraw at any time<br>without giving any reason, without my or my child's medical care or legal rights being affected.	☐	
3	I understand that by taking part in this study, my child will be randomly allocated to receive either 'treatment as usual' or 'Mentalization Based Treatment'.	☐	
4	I understand that personal information about my child and myself will remain confidential,<br>unless the researcher or clinician felt that I or another person were in danger of harm. In this<br>case, they may need to inform relevant agencies of this.	☐	
5	I understand that if I decide to withdraw, personal data I have provided up to that point cannot be deleted.	☐	
6	I understand that the CAMHS service treating will write to my child's GP to inform them that we are taking part in this research.	☐	
7	(optional) I agree to me and my child taking part in two online activities (about recognising emotions and talking about feelings), part of which will be video recorded.	☐	☐
8	(optional) I understand that the information collected about me will be used to support<br>other research in the future and may be shared anonymously with other researchers.	☐	☐
9	(optional) I agree to have an additional interview with a researcher to give feedback on my experience of therapy and of being part in this research	☐	☐

study. I agree that this interview can be video recorded.

10 (optional) I agree to be contacted in future about research following on from the ERiC study

11 I agree to take part in the above study, and for my child to take part.

______________________________ ______________________

Name of Parent/carer giving consent Date

Please tick here if you would like a copy of the signed consent form to be emailed to you
