# Supplementary material for: Emotion regulation in children (ERiC): A protocol for a randomised clinical trial to evaluate the clinical and cost effectiveness of Mentalization Based Treatment (MBT) vs Treatment as Usual for school-age children with mixed emotional and behavioural difficulties
Source: PLoS One. 2023 Aug 17;18(8):e0289503. doi: 10.1371/journal.pone.0289503 (PMC10434917; doi:10.1371/journal.pone.0289503)
Supplement: Peer review history

# Editor report: Decision Letter 0

**DOI:** 10.1371/journal.pone.0289503.r001

**Authors:** Vanessa Carels

20 Jul 2023

Emotion regulation in children (ERiC): A protocol for a randomised clinical trial to evaluate the clinical and cost effectiveness of Mentalization Based Treatment (MBT) for school-age children with mixed emotional and behavioural difficulties

PONE-D-23-19207

Dear Dr. Midgley,

We’re pleased to inform you that your manuscript has been judged scientifically suitable for publication and will be formally accepted for publication once it meets all outstanding technical requirements.

Within one week, you’ll receive an e-mail detailing the required amendments. When these have been addressed, you’ll receive a formal acceptance letter and your manuscript will be scheduled for publication.

An invoice for payment will follow shortly after the formal acceptance. To ensure an efficient process, please log into Editorial Manager at http://www.editorialmanager.com/pone/, click the 'Update My Information' link at the top of the page, and double check that your user information is up-to-date. If you have any billing related questions, please contact our Author Billing department directly at authorbilling@plos.org.

If your institution or institutions have a press office, please notify them about your upcoming paper to help maximize its impact. If they’ll be preparing press materials, please inform our press team as soon as possible -- no later than 48 hours after receiving the formal acceptance. Your manuscript will remain under strict press embargo until 2 pm Eastern Time on the date of publication. For more information, please contact onepress@plos.org.

Kind regards,

Vanessa Carels

Staff Editor

PLOS ONE

Journal Requirements:

1. We note that you have referenced (Unpublished) on page 42 which has currently not yet been accepted for publication. Please respond by return e-mail with a copy of your updated manuscript to include to remove this from your References and amend this to state in the body of your manuscript: (ie “Bewick et al. [Unpublished]”) as detailed online in our guide for authors

http://journals.plos.org/plosone/s/submission-guidelines#loc-reference-style.   We can then upload this to your submission on your behalf.

2. Please include your full ethics statement in the ‘Methods’ section of your manuscript file. In your statement, please include the full name of the IRB or ethics committee who approved or waived your study, as well as whether or not you obtained informed written or verbal consent. If consent was waived for your study, please include this information in your statement as well. 

# Editor report: Acceptance letter

**DOI:** 10.1371/journal.pone.0289503.r002

**Authors:** Vanessa Carels

8 Aug 2023

PONE-D-23-19207 

Emotion regulation in children (ERiC): A protocol for a randomised clinical trial to evaluate the clinical and cost effectiveness of Mentalization Based Treatment (MBT) vs Treatment as Usual for school-age... 

Dear Dr. Midgley:

I'm pleased to inform you that your manuscript has been deemed suitable for publication in PLOS ONE. Congratulations! Your manuscript is now with our production department. 

If your institution or institutions have a press office, please let them know about your upcoming paper now to help maximize its impact. If they'll be preparing press materials, please inform our press team within the next 48 hours. Your manuscript will remain under strict press embargo until 2 pm Eastern Time on the date of publication. For more information please contact onepress@plos.org.

If we can help with anything else, please email us at plosone@plos.org. 

Thank you for submitting your work to PLOS ONE and supporting open access. 

Kind regards, 

PLOS ONE Editorial Office Staff

on behalf of

Dr. Vanessa Carels 

Staff Editor

PLOS ONE
